# Collateral Damage in the Human Gut Microbiome - *Blastocystis* Is Significantly Less Prevalent in an Antibiotic-Treated Adult Population Compared to Non-Antibiotic Treated Controls

**DOI:** 10.3389/fcimb.2022.822475

**Published:** 2022-02-25

**Authors:** Ian B. Jeffery, Paul D. Cotter, Pauline D. Scanlan

**Affiliations:** ^1^ School of Microbiology, University College Cork, Cork, Ireland; ^2^ APC Microbiome Ireland, University College Cork, Cork, Ireland; ^3^ Teagasc Food Research Centre, Moorepark, Fermoy, Ireland

**Keywords:** blastocystis, gut microbiome, antibiotics, secondary extinctions, prevalence

## Abstract

Antibiotics can drive the rapid loss of non-target, phylogenetically diverse microorganisms that inhabit the human gut. This so-called “collateral damage” has myriad consequences for host health and antibiotic mediated changes to the gut microbiota have been implicated in the aetiology of many chronic diseases. To date, studies have largely focused on how antibiotics affect the bacterial fraction of the gut microbiome and their impact on non-bacterial members, including prevalent eukaryal species, such as *Blastocystis*, remains largely unknown. Here we assessed the prevalence and diversity of *Blastocystis* in an elderly adult group that were in receipt of antibiotics (n = 86) and an equivalent non-antibiotic treated group (n = 88) using a PCR-based approach. This analysis revealed that although similar subtypes were present in both groups, *Blastocystis* was significantly less prevalent in the antibiotic-treated group (16%) compared to non-antibiotic treated controls (55%); Fisher’s Exact test, *p* < 0.0001). Given that antibiotics target structures and molecules of prokaryotic cells to kill or inhibit bacterial populations, the most likely explanation for differences in prevalence between both groups is due to secondary extinctions owing to the potential dependence of *Blastocystis* on bacteria present in the gut microbiome that were negatively affected by antibiotic treatment. Although further work is required to explore this hypothesis in greater detail, these data clearly show that *Blastocystis* prevalence in human populations is negatively associated with antibiotic treatment. This finding may be relevant to explaining patterns of variation for this microorganism in different human populations and cohorts of interest.

## Introduction

Antibiotics are life-saving medicines that play a central role in the fight against infectious disease as well as facilitating many medical practices such as surgeries and chemotherapy (Hutchings et al., 2019). However, antibiotic treatments can drive the rapid loss of non-target, and phylogenetically and functionally diverse, microorganisms associated with the human body (Dethlefsen and Relman, 2011; Korpela et al., 2016; Elvers et al., 2020; Ramirez et al., 2020; Maier et al., 2021; Pennycook and Scanlan, 2021). The potential negative effects of this so-called “collateral damage” on the human microbiome has myriad implications for host health and has been attributed, in part, to the rise of many chronic immunological and metabolic disorders such as obesity and diabetes (Bailey et al., 2014; Boursi et al., 2015; Saari et al., 2015; Bejaoui and Poulsen, 2020). Moreover, in some scenarios, antibiotic administration can lead to extreme perturbations and loss of microbiota predisposing individuals to expansion of and/or invasion by opportunistic pathogens resulting in potentially fatal infections such as *Clostridioides difficile* associated disease (CDAD) (Spencer, 1998; Baxter et al., 2008). Accordingly, an understanding of how antibiotics affect the ecology and evolution on the human gut microbiota has been the subject of a multitude of different *in vitro, in silico* and *in vivo* studies (Dethlefsen and Relman, 2011; Candon et al., 2015; Shaw et al., 2019; Chng et al., 2020; Ramirez et al., 2020; Pennycook and Scanlan, 2021).

To date, these studies have largely focused on how antibiotics affect the bacterial fraction of the community, with some exceptions (Górska et al., 2018; Seelbinder et al., 2020; Haak et al., 2021). This focus is intuitive given that most antibiotics target specific features of bacterial cells, and bacterial populations predominate the human gut microbiome. Resulting data shows that for some individuals, bacterial communities can apparently fully recover to preintervention states and exhibit resilience, whereas in other individuals, the bacterial community remains altered from its base-line status over the longer-term (Dethlefsen and Relman, 2011; Roodgar et al., 2021). This observed inter-individual variation in response to treatment is likely due to the class of antibiotic used, the dose and frequency of dose, the initial composition of the microbiota as well as other host and environment related factors (Ng et al., 2019; Chng et al., 2020; Pennycook and Scanlan, 2021; Rashidi et al., 2021). Crucially, recent studies have shown that antibiotics can alter the intestinal mycobiome (fungal component of the community) (Seelbinder et al., 2020) and virome (Górska et al., 2018; Haak et al., 2021), which indicates that the collateral effects of antibiotics on the gut microbiota extend beyond the bacterial fraction of the community. Nonetheless, the extent to which antibiotics impact on the prevalence and diversity of ecologically and clinically relevant species of intestinal microbial protists, such as *Blastocystis*, remains largely unknown.

*Blastocystis* is a prevalent and diverse member of the human gut microbiota as well as many other animal hosts (Alfellani et al., 2013a; Alfellani et al., 2013b; Scanlan et al., 2014; Betts et al., 2018; Tito et al., 2019). In human populations, a diversity of different subtypes (species) (Stensvold et al., 2007; Stensvold and Clark, 2020) are commonly found, and its presence is associated with a higher microbial diversity as well as the presence of specific bacterial genera (Andersen et al., 2015; The Blastocystis Investigation Group et al., 2016; Tito et al., 2019). Although *Blastocystis* has been implicated in several intestinal and extra-intestinal diseases, *Blastocystis* is highly prevalent in asymptomatic carriers and the emerging consensus is that the majority of *Blastocystis* subtypes are human commensals (Scanlan, 2012; Andersen and Stensvold, 2016; Stensvold and van der Giezen, 2018). However, *in vitro* models have shown that specific subtype variants can activate a pro-inflammatory immune response, and possess pathogenic traits such as the ability to adhere to enterocytes and disrupt epithelial barrier function (Puthia et al., 2008; Ajjampur and Tan, 2016). As such, it is likely that a pathogenic role for *Blastocystis* in human health is linked to subtype and intra-subtype *Blastocystis* genetic diversity and other host related factors such as immune status; a scenario which is analogous for other species of microbes that can colonise the human gut (Kaper et al., 2004; Tenaillon et al., 2010). However, despite increasing research into the diversity and function of *Blastocystis* in human populations, there remains a limited understanding of its ecological and functional roles, including how it interacts with the human host and other members of the microbial community (Scanlan and Stensvold, 2013; Stensvold and van der Giezen, 2018; Deng et al., 2021). Moreover, we also have limited information on how the prevalence and diversity of *Blastocystis* is impacted upon by important variables such as antibiotics, which are well recognised to affect the diversity and composition of the bacterial fraction of the community. To redress this knowledge gap we used a PCR based approach to survey the prevalence and diversity of *Blastocystis* between an adult antibiotic-treated group (n = 86) and an equivalent non-antibiotic treated control group (n = 88).

## Methods

### Study Subjects

The two groups of individuals analysed in this study were a subset of the ELDERMET study which investigated the relationship between diet, gut bacteria, and health status in an elderly Irish cohort (> 65 years) (Claesson et al., 2011; Claesson et al., 2012). Our sample-set comprised elderly community dwelling adults that were in receipt of antibiotics or had taken antibiotics within one month of sample collection (ABX+ group, n= 86), and an equivalent group of elderly community dwelling adults that were not in receipt of antibiotics and had not received antibiotics for at least three months prior to the study start date (ABX- group, n=88). The average age and female to male ratios for ABX+ and ABX- groups, were 75.2 ± 6.1 and 73.0 ± 6.2 (mean ± s.d.) and 1:1.2 and 1:1.3, respectively, see **Supplementary Table 1**. Medical and clinical data was available for a subset of individuals in our dataset, and included current medical conditions and other clinically relevant data such as Mini Nutritional Assessment (MNA) Mini mental state exam (MMSE), Barthel score, and Functional Independence Measures (FIM) which were recorded as previously reported (Claesson et al., 2012). Although many individuals recorded no active disease at time of sampling, given the age of the subjects, many reported age-related illnesses. Where specific information relating to gastrointestinal disease or complaints is listed, only eight individuals (ABX-, n = 6 and ABX+, n=2) reported having any gastrointestinal complaints; three of these had diverticular disease, two reported IBS like symptoms, two reported dyspepsia, and another gastritis, full details on medical and clinical data are provided in **Supplementary Tables 2**, **3**. Where information is given as to why an individual was receiving antibiotics this is provided in **Supplementary Table 4**. Data on *Blastocystis* prevalence and diversity from the ABX- group was taken from two previously published studies (Scanlan et al., 2014; Scanlan et al., 2015), however, the prevalence and diversity data for the ABX+ group was collected during the same time. Individuals in the ABX+ group received different numbers of antibiotic treatments, as well as different classes of antibiotics, and further details for individuals where this information is available is listed in **Supplementary Table 4**.

### *Blastocystis* PCR and Sequence Analysis

Genomic DNA was extracted from faecal samples as described elsewhere (Claesson et al., 2011) and PCRs to assay for *Blastocystis* prevalence and perform allele barcoding were conducted using the primer pair RD5 and BhRDr according to standard protocol (Scicluna et al., 2006; Scanlan et al., 2014). Every sample was assayed in triplicate and a single positive from each individual was cleaned and sent for Sanger sequencing. *Blastocystis* subtype and allele was assigned using the online database http://pubmlst.org/blastocystis/. Sequences were then aligned and analysed in MEGA5 (Tamura et al., 2011). Additional PCRs assays were conducted to determine the diversity of different species or subtypes within and between individuals using nested PCRs and primers targeting ST1, ST2, ST3 and ST4 as outlined elsewhere (Scanlan et al., 2015).

### Bacterial Diversity and Taxonomic Analysis

Information on bacterial alpha diversity was calculated from a previously generated 16S rRNA gene dataset (Jeffery et al., 2016) that was available for a subset of individuals (n = 153). In brief DNAs were extracted from these faecal samples and the V4 region of the 16S rRNA gene was amplified and then sequenced on a 454 Genome Sequencer FLX Titanium platform (Roche Diagnostics and Beckman Coulter Genomics). The returned 454 sequence reads were pre-processed using a QIIME pipeline with parameters as previously reported (Jeffery et al., 2016). Raw sequence reads were quality trimmed using the QIIME pipeline with the following criteria: (1) two mismatches were allowed in barcode sequences; (2) reads could not begin with ambiguous bases (Ns); (3) the minimum average quality score was 25. The remaining criteria for quality trimming were as per default settings from QIIME’s split_libraries.py. Singletons and the identification and removal of chimeric sequences was performed using chimera.uchime from the Mothur project (Schloss et al., 2009). Sequences were then filtered by length (204–212 bases) and sequence reads were clustered into operational taxonomic units (OTUs, at 97% similarity) using two-stage clustering (Jiang et al., 2012). The following alpha diversity indices; Chao1, ACE, Shannon and Simpsons were calculated using the R library PhyloSeq with the OTU dataset rarefied to 5000 reads (McMurdie and Holmes, 2013). Genus level data was generated using the wang method for classify.seqs from the Mothur project with RDP trainset 9. The SPINGO methodology was used to classify the sequences to the species level and taxonomic classifications were filtered for a confidence value of greater than 0.6. (Allard et al., 2015).

### Statistical Analysis

We tested for any significant associations between our two groups of interest (*e.g.* ABX+ or ABX-) and *Blastocystis* prevalence using Fisher’s Exact tests for these nominal variables. We tested for a significant difference in time (days) from last antibiotic dose and faecal sample collection between *Blastocystis* positive and negative individuals within the ABX+ group using an unpaired t-test. We tested for a significant difference in the number of antibiotics treatments received per individual between *Blastocystis* positive and negative samples within the ABX+ group using a Mann-Whitney test. ABX- and ABX+ group were further sub-divided into ABX- *Blastocystis*-, ABX-*Blastocystis*+, ABX+*Blastocystis-*, and ABX+*Blastocystis*- to test for significant differences in mean bacterial alpha diversity, for all four measures of this index, between these four groups using ANOVA and Tukey’s HSD *post-hoc* test. These statistical analyses were carried out using SPSS version 28.0.0.0. Statistical analyses of the 16S rRNA dataset was performed using the DESeq2 methodology (Love et al., 2014) with adjustment for antibiotic usage.

## Results

### Prevalence of *Blastocystis* Is Reduced in Antibiotic Treated Group

**Figure 1:**
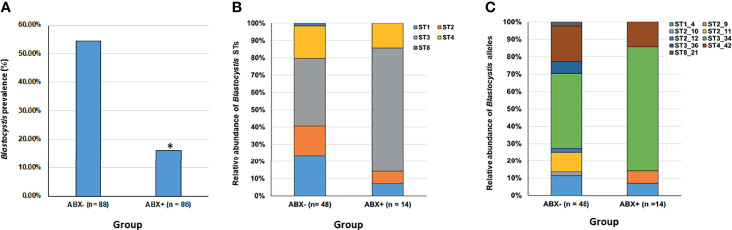
Overview of Blastocystis prevalence and diversity in an antibiotic-treated group compared to controls. **(A)** Blastocystis prevalence is significantly reduced in an antibiotic-treated group (ABX+) compared to controls (*Fisher’s Exact test, p < 0.0001). **(B)** Relative abundance of different Blastocystis subtypes (STs) within the antibiotic-treated group (ABX+) compared to controls (ABX-). **(C)** Relative abundance of different Blastocystis subtype allele variants within the antibiotic-treated group (ABX+) compared to controls (ABX-).

PCR analysis revealed that *Blastocystis* is significantly less prevalent in the antibiotic-treatment group compared to the control non-antibiotic treated group, Fisher’s Exact test *p* < 0.0001, see **Figure 1A**. 14 out of 86, or 16% of individuals were positive for *Blastocystis* in the ABX+ compared to 48 out of 88, or 55% in the ABX- control group.

### Diversity of *Blastocystis* in Antibiotic Treated and Non-Antibiotic Group

A total of five different STs were detected in our dataset, ST1, ST2, ST3, ST4 and ST8; except for ST8, these STs are the mostly commonly recovered STs in human populations. The relative abundance of STs between the two groups is presented in **Figure 1B** and it is evident that although similar STs are present in both groups there is slight variation in their relative abundance. There were eight “co-colonisations” evident in our ABX- group with only single ST colonisations detected in our ABX+ group. Except for co-colonisation positive cases that were detected using ST-specific PCRs (where there is insufficient sequence length to provide allele discrimination), we could provide allele information on STs present in both groups, see **Figure 1C**. Eight different ST intra-subtypes were detected in the ABX- group compared to four different ST intra-subtypes in the ABX+ group.

### No Significant Difference in Mean Time (Days) Between Antibiotic Treatment Date and Sample Collection, and Mean Number of Antibiotic Treatments Taken, for *Blastocystis* Positive and Negative Individuals Within the ABX+ Group

A recent antibiotic history was also available for a subset of individuals in the antibiotic treated group (n = 54) and is provided in **Supplementary Table 4**. There was no significant difference in time (days), between the date of last antibiotic taken and sample collection between individuals that were *Blastocystis* positive (ABX+/Blasto+; n = 10, mean 16.07) and negative in the ABX+ group (ABX+/Blasto-; n = 45, mean = 12.34), *p* = 0.250983. Similarly, there was no significant difference in the number of antibiotic treatments taken for *Blastocystis* positive (n =10, mean 3.70) and negative individuals (n = 45, mean 3.89) within the ABX+ group, *p* = 0.785.

### There Are Significant Differences in Bacterial Alpha Diversity Between *Blastocystis* Positive and Negative Subgroups and Significant Associations Between *Blastocystis* and Specific Bacterial Genera

Information on bacterial alpha diversity was calculated using an OTU dataset rarified to 5000 reads per sample from a previously generated 16S rRNA gene dataset (Jeffery et al., 2016) that was available for a subset of individuals. The results of this analysis is summarised in **Table 1** and full details are provided in **Supplementary Table 5**. We calculated four different measures of alpha diversity (Chao1, ACE, Shannon and Simpson). For all four alpha diversity indices there was a significant difference (*p* < 0.001) in mean bacterial alpha diversity between the four subgroups *e.g.* ABX-/*Blasto*- (n = 34), ABX-/*Blasto+* (n = 42), ABX+ *Blasto-* (n = 65), ABX+/*Blasto+* (n = 12), see **Table 1**. We observed the same trend in the running order of highest to lowest mean diversity as follows ABX-Blasto+ > ABX+Blasto+ > ABX-Blasto- > ABX+Blasto- for all indices with the exception of Shannon index which was as follows ABX-Blasto+ > ABX-Blasto- > ABX+Blasto+ > ABX+Blasto-. Further, Tukey’s HSD revealed significant pairwise differences (*p* < 0.05) between different subgroups based on alpha diversity measure, see **Table 1**.

**Table 1:** Mean and standard deviations of different measures of alpha diversity per antibiotic and *Blastocystis* grouping.

Alpha diversity Metric	Grouping				*F* statistic and *P* value
	ABX-Blasto- (n = 34)	ABX-Blasto+ (n = 42)	ABX+Blasto- (n = 65)	ABX+Blasto+ (n = 12)	
Chao1 (Mean ± SD)	255.4 ± 82.12*	309.62 ± 61.09*,°	234.35 ± 69.54°	261.3 ± 53.88	*F*_3, 148_ =10.137, *p* < 0.001
ACE (Mean ± SD)	254.03 ±. 79.91*	302.26 ± 52.5*,°	233.43 ± 66.64°	262.45 ± 57.2	*F*_3, 148_ = 9.415, *p* < 0.001
Shannon (Mean ± SD)	3.51 ± 0.529*°	3.86 ± 0.338*^♦♠^	3.24 ± 0.521°^♦^	3.44 ± 0 .37^♠^	*F*_3, 148_ = 15.245, *p* < 0.001
Simpson (Mean ± SD)	0.92 ± 0.051	0.95 ± 0.027*	0.9 ± 0.063*	0.93 ± 0.053	*F*_3, 148_ = 6.924, *p* < 0.001

Mean difference significance levels (p < 0.0.5) between antibiotic and Blastocystis prevalence subgroups for each calculated alpha diversity index are designated by shared symbols between groups e.g. *^,^°^,♦,♠^

To assess differences in microbiota composition between microbiota profiles with and without *Blastocystis* the DESeq2 methodology was applied with adjustment for antibiotic usage. The abundance of two bacterial genera, *Anaerosporobacter* and *Robinsoniella*, both members of the *Lachnospiraceae* family of bacteria, were positively correlated with *Blastocystis* irrespective of antibiotic status. Conversely, the following bacterial genera; *Bacteroides*, *Bilophila*, *Escherichia/Shigella, Parasutterella, Subdoligranulum, Clostridium_Xl*Vb and *Flavonifractor* were significantly decreased in *Blastocystis* positive individuals, see **Supplementary Table 6**.

## Discussion

Protists, such as *Blastocystis* are common in human populations and estimates indicate that over 1 billion people worldwide carry this microorganism (Andersen and Stensvold, 2016). Here we sought to address how antibiotic treatment, a key factor that is well-recognised to adversely impact on the diversity and composition of the human gut microbiome (Baxter et al., 2008; Dethlefsen and Relman, 2011; Ramirez et al., 2020), affects *Blastocystis* prevalence in adult populations. To do so, we used a PCR based approach to survey the prevalence and diversity of *Blastocystis* between an adult antibiotic-treated group (n = 86) and an equivalent non-antibiotic treated control group (n = 88). Although similar subtypes (STs) were present in both groups, our results clearly show that *Blastocystis* prevalence is significantly lower in the antibiotic-treated group compared to non-antibiotic controls.

Individuals in this study received one or more antibiotic treatments belonging to different antibiotic classes, see **Supplementary Table 4**. These different antibiotic classes could be further categorised into groups based on one of three different modes of action *e.g*. inhibition of cell wall synthesis, inhibition of protein synthesis and inhibition of nucleic acid synthesis. With the exception of three antimicrobials listed, it is clear that all antimicrobials in this study belong to classes that are deployed to target bacterial populations and their modes of action largely target features of bacterial cells that are absent in eukaryotes such as *Blastocystis* (*e.g.* cell wall synthesis and 30S and 50S ribosomes) (Kohanski et al., 2010; Kapoor et al., 2017). Consequently, it is unlikely that the antibiotics used in this study are directly killing *Blastocystis* populations, but rather the reduced prevalence observed in the ABX+ group is due to indirect effects of antibiotic treatment *i.e.* antibiotics are killing or inhibiting bacterial populations that *Blastocystis* potentially requires for colonisation, growth and persistence in the human gut. This phenomenon is known as secondary extinction and has been the focus of several empirical and theoretical studies, although primarily in macroecological and conservation-based studies of plants, animals, and insects (Brodie et al., 2014; Schleuning et al., 2016). Of note, secondary extinctions can occur for a range of different types of species interactions including mutualistic and antagonistic interactions, and the risk of secondary extinction is predicted to be high for species that cannot find new partners in communities that are reassembling following changes in environmental conditions (Eklöf and Ebenman, 2006; Brodie et al., 2014) - a scenario analogous to antibiotic mediated perturbation of the gut microbiota.

In support of our secondary extinction hypothesis, numerous studies have shown that *Blastocystis* is correlated with a higher microbial diversity and the presence of specific microbial taxa (Andersen et al., 2015; The Blastocystis Investigation Group et al., 2016; Tito et al., 2019). These data are largely consistent across different studies and most have shown positive correlations between *Blastocystis* carriage and *Ruminococcus* and *Prevotella*, and negative correlations between *Blastocystis* carriage and *Bacteroides* and *Proteobacteria* in adult populations (Andersen et al., 2015; The Blastocystis Investigation Group et al., 2016; Gabrielli et al., 2020). Detailed analysis of ST diversity has also shown inverse correlations between the presence of particular *Blastocystis* STs (ST3 and ST4) and specific bacterial taxa *e.g. Akkermansia* (Tito et al., 2019). Although these correlations could represent epiphenomena (*e.g.* certain diets or other factors could independently promote the growth of particular microbes), difficulties with axenising cultures of *Blastocystis* (Zierdt, 1991), the presence of bacteria on *Blastocystis* surfaces (Cassidy et al., 1994), together with changes in microbiota composition following *Blastocystis* colonisation in animal models (Billy et al., 2021) and co-coculture experiments showcasing positive and negative effects of *Blastocystis* on bacterial populations (Yason et al., 2019) provide compelling evidence for direct interactions between *Blastocystis* and bacteria. Here, our analyses show that there are significant differences in mean bacterial alpha diversity for our four subgroups with the highest mean bacterial alpha diversity observed for the ABX-/*Blastocystis+* subgroup compared to all other three subgroups, irrespective of the index used, see **Table 1**. With the exception of the Shannon index of alpha diversity mean diversity is also higher in ABX+/*Blastocystis+* compared to ABX-/*Blastocystis*- and ABX+/*Blastocystis*- subgroups. These results are consistent with data from aforementioned studies showing that *Blastocystis* carriage is linked to a higher bacterial diversity, and further show that *Blastocystis* prevalence is linked to a significantly higher mean bacterial alpha diversity when comparing within our antibiotic treated group and with our *Blastocystis* negative ABX- controls. Further, taxonomic analysis revealed significant associations between the presence of *Blastocystis* and specific bacterial genera, irrespective of antibiotic grouping. Notably *Bacteroides* and members of the Proteobacteria, such as *Escherichia/Shigella and Parasutterella* were decreased in *Blastocystis* positive individuals and *Anaerosporobacter* and *Robinsoniella*, both members of the *Lachnospiraceae* family within the Firmicutes phyla, were positively correlated with *Blastocystis*. Given that these data are largely consistent with previously reported findings from independent studies (Deng et al., 2021), this re-enforces the idea that there are strong links between the potential presence/absence of *Blastocystis* and the composition of the gut microbiota.

A diversity of different *Blastocystis* STs and intra-subtype variants were recovered in this study. We did not detect any co-colonisations in our ABX+ group and there is also a reduced diversity of intra-subtypes present in this group. However, this latter finding may be due to the lower number of positive cases in the ABX+ group rather than certain intra-subtypes being more susceptible to the potential indirect effects of antibiotic treatment. Greater sample sizes of antibiotic treated populations, with potentially higher positive *Blastocystis* cases, could help shed further light on this.

As already outlined individuals in the ABX+ group were in receipt of a range of different antibiotic treatments prior to and/or at the time of sampling that could be grouped based on mode of action. Twenty-seven individuals were in receipt of cell wall synthesis inhibiting (CWI) antibiotics, 1 individual was in receipt of nucleic acid synthesis (NAI) inhibiting antibiotics, and 5 individuals were in receipt of protein synthesis inhibiting (PSI) antibiotics. The remaining individuals in the ABX+ group were in receipt of two or more types of antibiotic groups as follows; CWI and NAI antibiotics, n = 10, CWI and PSI, n= 6, NAI and PSI, n = 1, and finally 4 individuals were in receipt of all three antibiotic groups e.g. CWI, PSI and NAI classes. The breakdown of these groupings together with the number and timing of treatments individuals received are outlined in **Supplementary Table 4**. Unfortunately, due to the low number of *Blastocystis* positive and negative cases for some of these antibiotic class groupings and combinations of groupings, this particular set of data does not lend itself to statistical analysis, however, it is clear that all bar seven individuals were either receiving or had recently received an antibiotic that targeted the bacterial cell wall at the time of sample collection. Nevertheless, we did have sufficient positive and negative cases to look at the mean difference in number of treatments taken per individual and the mean difference in time (days) between the date of last antibiotic treatment and sample collection for those who were *Blastocystis* positive and negative within the ABX+ group. Interestingly we found no significant difference in the mean number of treatments taken between individuals that were *Blastocystis* positive and negative within the ABX+ group. We also did not find any significant difference in time between the date of last antibiotic treatment and sample collection between positive and negative individuals within the ABX+ group. These data are important, as they are linked to how microbiomes are affected by and recover following antibiotic perturbation. However, there is considerable inter-individual variation in both the effect of antibiotics on bacterial populations in the gut microbiome, as well as their ability to recover, and this variability in response is due to a range of factors including initial microbiota composition (Dethlefsen and Relman, 2011; Ng et al., 2019; Chng et al., 2020; Pennycook and Scanlan, 2021). This, taken together with the lack of significant effect of treatment number, and time since treatment, on prevalence rates, as well as the fact that there are some positive cases within the ABX+ group, suggests that antibiotic treatment also has differential effects on *Blastocystis* carriage at the individual level. For example, for *Blastocystis* positive individual’s antibiotic treatment may have had no effect on the bacterial populations that it depends upon for survival in the gut (mean alpha diversity is significantly higher for ABX+/*Blastocystis*+ subgroup than ABX+/*Blastocystis*- and ABX-/*Blastocystis*- subgroups). For others, where *Blastocystis* was potentially affected by antibiotic treatment perhaps insufficient time had elapsed for their microbiome to recover to baseline to facilitate *Blastocystis* to recolonise. Alternatively, their microbiome may have recovered to an alternative state that did not facilitate recolonisation. The basis of these arguments centre on the idea that inter-kingdom interactions between bacteria and *Blastocystis* is the primary factor underpinning *Blastocystis* prevalence rates in the human gut, and as outlined there is strong data to support this assertion. However, the genetic and phenotypic diversity of *Blastocystis* is likely considerable (Gentekaki et al., 2017) and therefore it is important to consider that not every *Blastocystis* population in the human gut may be dependent on bacterial diversity and composition. Moreover, we cannot entirely rule out that antibiotics could have cytotoxic or inhibitory effects on *Blastocystis* as protein and DNA synthesis are universal processes and recent data has shown that aminoglycosides can interact with the eukaryotic 80S subunit (Prokhorova et al., 2017) and certain antibiotics such as tetracyclines can interfere with mitochondrial function (Moullan et al., 2015). Of note, precedence for the potential importance of antibiotics as mediators of *Blastocystis* secondary extinctions exists in the literature. In a study from 1999, the combination of two antibiotics, Trimethoprim (DNA synthesis inhibition) and Sulfamethaxazole (folic acid synthesis inhibition), that are used to treat a wide range of bacterial infections, resulted in *Blastocystis* clearance rates of 94.3% in positive individuals (n = 53) (Ok et al., 1999). Similar to what we report here, the authors concluded that the “drug may directly affect *B. hominis* or it may act by destroying the bacterial flora necessary for its growth, or both (Ok et al., 1999)”.

## Conclusions

Understanding how the human gut microbiota is affected by antibiotics is not only fundamental to our understanding of how microbiomes are structured and respond to perturbation, but also central to our understanding of how antibiotic mediated effects on the microbiota impact host health. Here, we show that *Blastocystis* is significantly less prevalent in an antibiotic treated group compared to non-treated controls. Although we do not have a precise explanation for this finding, the most likely reason is that reduced prevalence is due to the secondary extinction of *Blastocystis*, at least for some individuals. Although further work is required to better understand how *Blastocystis* interacts with and potentially depends on specific bacteria for colonisation and persistence in the human gut, secondary extinctions can occur in both mutualistic and antagonistic interactions and occur following ecosystem perturbation.

## Data Availability Statement

The original contributions presented in the study are included in the article/**Supplementary Material**. Further inquiries can be directed to the corresponding author.

## Ethics Statement

The studies involving human participants were reviewed and approved by Cork Clinical Research Ethics Committee. The patients/participants provided their written informed consent to participate in this study.

## Author Contributions

PS conceived the study, performed the experiments and data analysis, and wrote the paper. IJ and PC performed data analysis and edited the manuscript. All authors contributed to the article and approved the submitted version.

## Funding

This research was funded by a Marie Curie Intra-European Fellowship to PS (Grant No. 328673).

## Conflict of Interest

The authors declare that the research was conducted in the absence of any commercial or financial relationships that could be construed as a potential conflict of interest.

## Publisher’s Note

All claims expressed in this article are solely those of the authors and do not necessarily represent those of their affiliated organizations, or those of the publisher, the editors and the reviewers. Any product that may be evaluated in this article, or claim that may be made by its manufacturer, is not guaranteed or endorsed by the publisher.
