# Supplementary material for: Collateral Damage in the Human Gut Microbiome - Blastocystis Is Significantly Less Prevalent in an Antibiotic-Treated Adult Population Compared to Non-Antibiotic Treated Controls
Source: Front Cell Infect Microbiol. 2022 Feb 25;12:822475. doi: 10.3389/fcimb.2022.822475 (PMC8913940; doi:10.3389/fcimb.2022.822475)
Supplement: Supplementary file 1 [file Table_1.docx]

**Supplementary Table 1.** Overview of study participants, treatment groups, *Blastocystis* prevalence and diversity

| **Sample Number** | **Antibiotic Group** | **Age** | **Gender** | ***Blastocystis* PCR result** | **Subtype** | **Allele** | **Co-colonisations (additional STs detected)** |
| --- | --- | --- | --- | --- | --- | --- | --- |
| EM1 | ABX- | 69 | Female | Negative | 0 | 0 | No |
| EM2 | ABX- | 77 | Female | Negative | 0 | 0 | No |
| EM3 | ABX- | 72 | Female | Negative | 0 | 0 | No |
| EM4 | ABX- | 72 | Male | Negative | 0 | 0 | No |
| EM5 | ABX- | 76 | Male | Positive | 3 | 34 | No |
| EM6 | ABX- | 71 | Female | Positive | 3 | 34 | No |
| EM7 | ABX- | 74 | Male | Positive | 4 | 42 | No |
| EM8 | ABX- | 68 | Male | Negative | 0 | 0 | No |
| EM9 | ABX- | 80 | Female | Negative | 0 | 0 | No |
| EM10 | ABX- | 75 | Female | Positive | 3 | 34 | No |
| EM11 | ABX- | 72 | Female | Negative | 0 | 0 | No |
| EM12 | ABX- | 74 | Female | Positive | 3 | 36 | No |
| EM13 | ABX- | 77 | Male | Positive | 3 | 34 | No |
| EM14 | ABX- | 66 | Male | Positive | 4 | 42 | No |
| EM15 | ABX- | 67 | Male | Negative | 0 | 0 | No |
| EM16 | ABX- | 85 | Male | Negative | 0 | 0 | No |
| EM17 | ABX- | 85 | Female | Positive | 2 | 10 | No |
| EM18 | ABX- | 65 | Female | Positive | 1 | 4 | No |
| EM19 | ABX- | 72 | Female | Positive | 2 | 11 | No |
| EM20 | ABX- | 78 | Male | Negative | 0 | 0 | No |
| EM21 | ABX- | 73 | Female | Positive | 3 | 36 | No |
| EM22 | ABX- | 72 | Female | Negative | 0 | 0 | No |
| EM23 | ABX- | 67 | Male | Positive | 3 | 34 | No |
| EM24 | ABX- | 66 | Male | Positive | 1 | 4 | Yes (ST4) |
| EM25 | ABX- | 73 | Female | Positive | 4 | 42 | No |
| EM26 | ABX- | 77 | Male | Negative | 0 | 0 | No |
| EM27 | ABX- | 72 | Male | Positive | 3 | 34 | No |
| EM28 | ABX- | 65 | Male | Negative | 0 | 0 | No |
| EM29 | ABX- | 72 | Male | Negative | 0 | 0 | No |
| EM30 | ABX- | 67 | Female | Positive | 3 | 34 | No |
| EM31 | ABX- | 65 | Male | Negative | 0 | 0 | No |
| EM32 | ABX- | 67 | Female | Negative | 0 | 0 | No |
| EM33 | ABX- | 72 | Female | Negative | 0 | 0 | No |
| EM34 | ABX- | 72 | Male | Negative | 0 | 0 | No |
| EM35 | ABX- | 73 | Male | Negative | 0 | 0 | No |
| EM36 | ABX- | 70 | Male | Positive | 2 | 11 | Yes (ST1 and ST3) |
| EM37 | ABX- | 69 | Female | Positive | 1 | 4 | No |
| EM38 | ABX- | 77 | Female | Negative | 0 | 0 | No |
| EM39 | ABX- | 65 | Male | Positive | 3 | 34 | Yes (ST1) |
| EM40 | ABX- | 79 | Male | Positive | 3 | 34 | No |
| EM41 | ABX- | 78 | Male | Positive | 2 | 11 | No |
| EM42 | ABX- | 85 | Male | Positive | 4 | 42 | No |
| EM43 | ABX- | 74 | Male | Negative | 0 | 0 | No |
| EM44 | ABX- | 71 | Male | Negative | 0 | 0 | No |
| EM45 | ABX- | 65 | Male | Positive | 3 | 36 | No |
| EM46 | ABX- | 93 | Male | Negative | 0 | 0 | No |
| EM47 | ABX- | 68 | Female | Positive | 4 | 42 | No |
| EM48 | ABX- | 83 | Male | Negative | 0 | 0 | No |
| EM49 | ABX- | 85 | Male | Negative | 0 | 0 | No |
| EM50 | ABX- | 67 | Male | Positive | 3 | 34 | No |
| EM51 | ABX- | 66 | Female | Positive | 4 | 42 | No |
| EM52 | ABX- | 70 | Female | Negative | 0 | 0 | No |
| EM53 | ABX- | 69 | Female | Positive | 4 | 42 | No |
| EM54 | ABX- | 71 | Male | Positive | 3 | 34 | No |
| EM55 | ABX- | 64 | Female | Negative | 0 | 0 | No |
| EM56 | ABX- | 77 | Female | Positive | 3 | 34 | No |
| EM57 | ABX- | 74 | Female | Positive | 3 | 34 | No |
| EM58 | ABX- | 68 | Female | Positive | 3 | 34 | No |
| EM59 | ABX- | 73 | Male | Positive | 3 | 34 | No |
| EM60 | ABX- | 70 | Male | Negative | 0 | 0 | No |
| EM61 | ABX- | 75 | Female | Negative | 0 | 0 | No |
| EM62 | ABX- | 81 | Male | Negative | 0 | 0 | No |
| EM63 | ABX- | 78 | Female | Positive | 2 | 9 | Yes (ST1) |
| EM64 | ABX- | 81 | Female | Negative | 0 | 0 | No |
| EM65 | ABX- | 69 | Female | Positive | 1 | 4 | No |
| EM66 | ABX- | 69 | Female | Negative | 0 | 0 | No |
| EM67 | ABX- | 71 | Female | Negative | 0 | 0 | No |
| EM68 | ABX- | 67 | Female | Positive | 2 | 9 | Yes (ST1) |
| EM69 | ABX- | 79 | Female | Negative | 0 | 0 | No |
| EM70 | ABX- | 81 | Female | Positive | 3 | 34 | No |
| EM71 | ABX- | 76 | Female | Negative | 0 | 0 | No |
| EM72 | ABX- | 74 | Female | Positive | 2 | 9 | Yes (ST1) |
| EM73 | ABX- | 76 | Male | Positive | 2 | 11 | No |
| EM74 | ABX- | 68 | Male | Negative | 0 | 0 | No |
| EM75 | ABX- | 65 | Female | Positive | 3 | 34 | Yes (ST1) |
| EM76 | ABX- | 69 | Male | Positive | 3 | 34 | Yes (ST1) |
| EM77 | ABX- | 86 | Female | Positive | 8 | 21 | No |
| EM78 | ABX- | 81 | Male | Positive | 1 | 4 | No |
| EM79 | ABX- | 79 | Female | Positive | 3 | 34 | No |
| EM80 | ABX- | 67 | Female | Negative | 0 | 0 | No |
| EM81 | ABX- | 67 | Female | Negative | 0 | 0 | No |
| EM82 | ABX- | 78 | Female | Positive | 4 | 42 | No |
| EM83 | ABX- | 71 | Female | Negative | 0 | 0 | No |
| EM84 | ABX- | 70 | Female | Negative | 0 | 0 | No |
| EM85 | ABX- | 64 | Female | Positive | 2 | 9 | No |
| EM86 | ABX- | 86 | Female | Positive | 2 | 11 | No |
| EM87 | ABX- | 70 | Male | Positive | 2 | 12 | No |
| EM88 | ABX- | 65 | Female | Positive | 4 | 42 | No |
| EM_ABX_1 | ABX+ | 71 | Male | Negative | 0 | 0 | No |
| EM_ABX_2 | ABX+ | 77 | Male | Negative | 0 | 0 | No |
| EM_ABX_3 | ABX+ | 71 | Female | Negative | 0 | 0 | No |
| EM_ABX_4 | ABX+ | 68 | Female | Negative | 0 | 0 | No |
| EM_ABX_5 | ABX+ | 76 | Male | Positive | 3 | 34 | No |
| EM_ABX_7 | ABX+ | 70 | Male | Positive | 4 | 42 | No |
| EM_ABX_8 | ABX+ | 66 | Female | Negative | 0 | 0 | No |
| EM_ABX_9 | ABX+ | 67 | Female | Negative | 0 | 0 | No |
| EM_ABX_10 | ABX+ | 68 | Female | Negative | 0 | 0 | No |
| EM_ABX_11 | ABX+ | 67 | Female | Negative | 0 | 0 | No |
| EM_ABX_12 | ABX+ | 78 | Female | Negative | 0 | 0 | No |
| EM_ABX_13 | ABX+ | 74 | Male | Negative | 0 | 0 | No |
| EM_ABX_14 | ABX+ | 82 | Female | Negative | 0 | 0 | No |
| EM_ABX_15 | ABX+ | 73 | Female | Positive | 1 | 4 | No |
| EM_ABX_16 | ABX+ | 85 | Female | Negative | 0 | 0 | No |
| EM_ABX_17 | ABX+ | 77 | Male | Negative | 0 | 0 | No |
| EM_ABX_18 | ABX+ | 81 | Female | Positive | 3 | 34 | No |
| EM_ABX_19 | ABX+ | 70 | Female | Positive | 3 | 34 | No |
| EM_ABX_20 | ABX+ | 79 | Female | Positive | 3 | 34 | No |
| EM_ABX_21 | ABX+ | 75 | Male | Negative | 0 | 0 | No |
| EM_ABX_22 | ABX+ | 86 | Female | Negative | 0 | 0 | No |
| EM_ABX_23 | ABX+ | 82 | Female | Negative | 0 | 0 | No |
| EM_ABX_24 | ABX+ | 78 | Female | Negative | 0 | 0 | No |
| EM_ABX_25 | ABX+ | 76 | Female | Negative | 0 | 0 | No |
| EM_ABX_26 | ABX+ | 78 | Female | Negative | 0 | 0 | No |
| EM_ABX_27 | ABX+ | 76 | Male | Negative | 0 | 0 | No |
| EM_ABX_28 | ABX+ | 69 | Male | Negative | 0 | 0 | No |
| EM_ABX_29 | ABX+ | 71 | Female | Negative | 0 | 0 | No |
| EM_ABX_30 | ABX+ | 67 | Male | Negative | 0 | 0 | No |
| EM_ABX_31 | ABX+ | 65 | Female | Positive | 3 | 34 | No |
| EM_ABX_32 | ABX+ | 77 | Female | Negative | 0 | 0 | No |
| EM_ABX_33 | ABX+ | 83 | Female | Negative | 0 | 0 | No |
| EM_ABX_34 | ABX+ | 70 | Female | Negative | 0 | 0 | No |
| EM_ABX_35 | ABX+ | 88 | Male | Negative | 0 | 0 | No |
| EM_ABX_36 | ABX+ | 66 | Female | Negative | 0 | 0 | No |
| EM_ABX_37 | ABX+ | 73 | Male | Negative | 0 | 0 | No |
| EM_ABX_38 | ABX+ | 67 | Female | Positive | 2 | 9 | No |
| EM_ABX_39 | ABX+ | 70 | Female | Negative | 0 | 0 | No |
| EM_ABX_40 | ABX+ | 74 | Female | Negative | 0 | 0 | No |
| EM_ABX_41 | ABX+ | 84 | Female | Negative | 0 | 0 | No |
| EM_ABX_42 | ABX+ | 74 | Male | Negative | 0 | 0 | No |
| EM_ABX_43 | ABX+ | 74 | Female | Negative | 0 | 0 | No |
| EM_ABX_44 | ABX+ | 86 | Female | Negative | 0 | 0 | No |
| EM_ABX_45 | ABX+ | 79 | Male | Negative | 0 | 0 | No |
| EM_ABX_46 | ABX+ | 78 | Female | Negative | 0 | 0 | No |
| EM_ABX_47 | ABX+ | 77 | Female | Negative | 0 | 0 | No |
| EM_ABX_48 | ABX+ | 77 | Male | Positive | ST4 | 42 | No |
| EM_ABX_49 | ABX+ | 75 | Male | Negative | 0 | 0 | No |
| EM_ABX_50 | ABX+ | 75 | Male | Negative | 0 | 0 | No |
| EM_ABX_51 | ABX+ | 66 | Female | Negative | 0 | 0 | No |
| EM_ABX_52 | ABX+ | 77 | Female | Negative | 0 | 0 | No |
| EM_ABX_53 | ABX+ | 67 | Male | Negative | 0 | 0 | No |
| EM_ABX_54 | ABX+ | 78 | Male | Negative | 0 | 0 | No |
| EM_ABX_55 | ABX+ | 68 | Male | Negative | 0 | 0 | No |
| EM_ABX_56 | ABX+ | 73 | Male | Positive | ST3 | 34 | No |
| EM_ABX_57 | ABX+ | 76 | Male | Negative | 0 | 0 | No |
| EM_ABX_58 | ABX+ | 78 | Male | Negative | 0 | 0 | No |
| EM_ABX_59 | ABX+ | 81 | Female | Negative | 0 | 0 | No |
| EM_ABX_60 | ABX+ | 82 | Male | Negative | 0 | 0 | No |
| EM_ABX_61 | ABX+ | 69 | Male | Negative | 0 | 0 | No |
| EM_ABX_62 | ABX+ | 71 | Male | Negative | 0 | 0 | No |
| EM_ABX_63 | ABX+ | 77 | Female | Negative | 0 | 0 | No |
| EM_ABX_64 | ABX+ | 69 | Female | Negative | 0 | 0 | No |
| EM_ABX_65 | ABX+ | 77 | Male | Negative | 0 | 0 | No |
| EM_ABX_66 | ABX+ | 76 | Female | Negative | 0 | 0 | No |
| EM_ABX_67 | ABX+ | 73 | Male | Negative | 0 | 0 | No |
| EM_ABX_68 | ABX+ | 70 | Female | Negative | 0 | 0 | No |
| EM_ABX_69 | ABX+ | 90 | Female | Negative | 0 | 0 | No |
| EM_ABX_70 | ABX+ | 72 | Female | Negative | 0 | 0 | No |
| EM_ABX_71 | ABX+ | 65 | Male | Positive | ST3 | 34 | No |
| EM_ABX_72 | ABX+ | 74 | Female | Positive | ST3 | 34 | No |
| EM_ABX_73 | ABX+ | 80 | Male | Negative | 0 | 0 | No |
| EM_ABX_74 | ABX+ | 78 | Male | Positive | ST3 | 34 | No |
| EM_ABX_75 | ABX+ | 69 | Male | Negative | 0 | 0 | No |
| EM_ABX_76 | ABX+ | 69 | Male | Negative | 0 | 0 | No |
| EM_ABX_77 | ABX+ | 89 | Female | Negative | 0 | 0 | No |
| EM_ABX_78 | ABX+ | 87 | Female | Negative | 0 | 0 | No |
| EM_ABX_79 | ABX+ | 75 | Female | Negative | 0 | 0 | No |
| EM_ABX_80 | ABX+ | 82 | Male | Negative | 0 | 0 | No |
| EM_ABX_81 | ABX+ | 65 | Female | Negative | 0 | 0 | No |
| EM_ABX_82 | ABX+ | 79 | Male | Negative | 0 | 0 | No |
| EM_ABX_83 | ABX+ | 78 | Male | Negative | 0 | 0 | No |
| EM_ABX_84 | ABX+ | 76 | Male | Negative | 0 | 0 | No |
| EM_ABX_85 | ABX+ | 83 | Male | Negative | 0 | 0 | No |
| EM_ABX_86 | ABX+ | 75 | Male | Positive | ST3 | 34 | No |
| EM_ABX_87 | ABX+ | 78 | Male | Negative | 0 | 0 | No |

Codes: ABX- = control group, ABX+ antibiotic treated group, NA = Not available
