# Supplementary material for: Collateral Damage in the Human Gut Microbiome - Blastocystis Is Significantly Less Prevalent in an Antibiotic-Treated Adult Population Compared to Non-Antibiotic Treated Controls
Source: Front Cell Infect Microbiol. 2022 Feb 25;12:822475. doi: 10.3389/fcimb.2022.822475 (PMC8913940; doi:10.3389/fcimb.2022.822475)
Supplement: Supplementary file 2 [file Table_2.docx]

**Supplementary Table 2.** Overview of medical data available for community dwelling elderly adult groups

| **Sample Number** | ***Blastocystis* PCR result** | **Medical: Current condition 1** | **Medical: Current condition 2** | **Medical: Current condition 3** | **Medical: Current condition 4** | **Medical: Current condition 5** | **Medical: Current condition 6** | **Medical: Current condition 7** |
| --- | --- | --- | --- | --- | --- | --- | --- | --- |
| EM1 | Negative | Chronic inflammatory demyelinating polyradicular neuropathies |  |  |  |  |  |  |
| EM2 | Negative | Microcytic Anaemia | Hypertension | Ischaemic Heart Disease | Cervical Spondylosis | Hiatus Hernia | Macular Degeneration | Diverticular Disease |
| EM3 | Negative | Polymyalgia Rheumatica | Hypertension | Peripheral Vascular Disease | Osteoarthritis- Aches& Pains | Large lipoma abdominal wall- awaiting surgery |  |  |
| EM4 | Negative | Early dementia | Dupytrens syndrome (both hands and fingers) |  |  |  |  |  |
| EM5 | Positive | Mild-moderate Parkinson’s disease |  |  |  |  |  |  |
| EM6 | Positive | High Cholesterol |  |  |  |  |  |  |
| EM7 | Positive | Dysarthria / 7th nerve palsy due to infarction (July 2008) | pain in left hip - awaiting hip replacement |  |  |  |  |  |
| EM8 | Negative | Hypertension | Intermittent Claudication |  |  |  |  |  |
| EM9 | Negative | recurrent 'drop attacks' - no underlying pathology identified | anxiety / low mood | osteoarthritis of hands | Diverticular disease |  |  |  |
| EM10 | Positive | Chronic constipation x yrs - recent colonoscopy to investigate - no abnormalities detected |  |  |  |  |  |  |
| EM11 | Negative | Asthma x 20 Yrs | Arthritis Right Knee |  |  |  |  |  |
| EM12 | Positive | Osteopenia |  |  |  |  |  |  |
| EM13 | Positive | Mild Cognitive Impairment (Short Forgetfulness) | Raised Cholesterol - Treated | Benign Diverticular Disease diagnosed by Colonoscopy | Hypertension -Controlled |  |  |  |
| EM14 | Positive | No current active problem |  |  |  |  |  |  |
| EM15 | Negative | Helicobacter - unable to confirm |  |  |  |  |  |  |
| EM16 | Negative | Unsteady gait due to postural hypotension |  |  |  |  |  |  |
| EM17 | Positive | Pain in both knees |  |  |  |  |  |  |
| EM18 | Positive | No current active problems |  |  |  |  |  |  |
| EM19 | Positive | Oedema of legs |  |  |  |  |  |  |
| EM20 | Negative | Hypertension | Recurrent gout | Small incisional hernia with lower abdominal scar |  |  |  |  |
| EM21 | Positive | Hypercholesteremia |  |  |  |  |  |  |
| EM22 | Negative | Osteopenia | Hypertension | Hypercholesteremia | Osteoporosis |  |  |  |
| EM23 | Positive | Subject reports irritable bowel type symptoms - episode every 2 / 3 months, diarrhoea alternating with constipation and heartburn |  |  |  |  |  |  |
| EM24 | Positive | no active medical problems at time of visit | Subject cycles ~3 miles / day and used to jog every day |  |  |  |  |  |
| EM25 | Positive | Hypertension (10 years) | Hypercholesteremia (2 years) | Atrial fibrillation (2 years) | Arthritis |  |  |  |
| EM26 | Negative | Orthostatic hypotension (October 2008) |  |  |  |  |  |  |
| EM27 | Positive | No current active problems |  |  |  |  |  |  |
| EM28 | Negative | Haemorrhoids (December 2008) |  |  |  |  |  |  |
| EM29 | Negative | No active medical problems at time of visit |  |  |  |  |  |  |
| EM30 | Positive | No active medical problems at time of visit |  |  |  |  |  |  |
| EM31 | Negative | No active medical problems at time of visit |  |  |  |  |  |  |
| EM32 | Negative | Left sided abdominal pain. Query diverticular disease / query urinary tract infection | Long history of bowel problems, loose stool alternating with constipation (IBS like symptoms) |  |  |  |  |  |
| EM33 | Negative | Hypertension |  |  |  |  |  |  |
| EM34 | Negative | No current active problems |  |  |  |  |  |  |
| EM35 | Negative | No current active problems |  |  |  |  |  |  |
| EM36 | Positive | No current active problems |  |  |  |  |  |  |
| EM37 | Positive | No current active problems |  |  |  |  |  |  |
| EM38 | Negative | Osteo-arthritis |  |  |  |  |  |  |
| EM39 | Positive | Hypertension |  |  |  |  |  |  |
| EM40 | Positive | Hypertension | Hypercholesterolaemia |  |  |  |  |  |
| EM41 | Positive | Hypertension | Raised Cholesterol (unable to take statins. takes plant sterols daily) | Query - Macular Degeneration of eyes |  |  |  |  |
| EM42 | Positive | No current active problems |  |  |  |  |  |  |
| EM43 | Negative | Type 2 diabetes |  |  |  |  |  |  |
| EM44 | Negative | recurrent unexplained falls |  |  |  |  |  |  |
| EM45 | Positive | Hypertension x 3 yrs |  |  |  |  |  |  |
| EM46 | Negative | unexplained iron deficiency anaemia |  |  |  |  |  |  |
| EM47 | Positive | Elevated Cholesterol | Eczema / Psoriatic Skin Disorder (lifelong history mild form) | Tinnitus for years some loss of hearing left ear | Bladder Prolapse Ring inserted Feb2009 | Calf Pain on/off not investigated |  |  |
| EM48 | Negative | No current active problems | - |  |  |  |  |  |
| EM49 | Negative | Chronic kidney disease (stage 4) | hydronephrosis left kidney |  |  |  |  |  |
| EM50 | Positive | No current active problems | - |  |  |  |  |  |
| EM51 | Positive | Elevated Cholesterol- on treatment | Large wart like growth on right side of abdomen. (had it removed previously) |  |  |  |  |  |
| EM52 | Negative | No current active medical problem |  |  |  |  |  |  |
| EM53 | Positive | No current active medical problem |  |  |  |  |  |  |
| EM54 | Positive | No current active medical problem |  |  |  |  |  |  |
| EM55 | Negative | shortness of breath | Anxiety |  |  |  |  |  |
| EM56 | Positive | No current active medical problem |  |  |  |  |  |  |
| EM57 | Positive | No current active medical problem |  |  |  |  |  |  |
| EM58 | Positive | No current active medical problem |  |  |  |  |  |  |
| EM59 | Positive | Parkinson’s Disease |  |  |  |  |  |  |
| EM60 | Negative | Type 2 diabetes Mellitus |  |  |  |  |  |  |
| EM61 | Negative | No current active medical problem |  |  |  |  |  |  |
| EM62 | Negative | No current active medical problem |  |  |  |  |  |  |
| EM63 | Positive | No current active medical problem |  |  |  |  |  |  |
| EM64 | Negative | No current active medical problem |  |  |  |  |  |  |
| EM65 | Positive | No current active medical problem |  |  |  |  |  |  |
| EM66 | Negative | macular degeneration |  |  |  |  |  |  |
| EM67 | Negative | Primary hypothyroidism |  |  |  |  |  |  |
| EM68 | Positive | No current active medical problem |  |  |  |  |  |  |
| EM69 | Negative | Depression for previous 2 months |  |  |  |  |  |  |
| EM70 | Positive | Trigeminal neuralgia x 34 years |  |  |  |  |  |  |
| EM71 | Negative | No current active medical problem |  |  |  |  |  |  |
| EM72 | Positive | No current active medical problem |  |  |  |  |  |  |
| EM73 | Positive | No current active medical problem |  |  |  |  |  |  |
| EM74 | Negative | No current active medical problem |  |  |  |  |  |  |
| EM75 | Positive | No current active medical problem |  |  |  |  |  |  |
| EM76 | Positive | No current active medical problem |  |  |  |  |  |  |
| EM77 | Positive | Rheumatoid arthritis |  |  |  |  |  |  |
| EM78 | Positive | No current active medical problem |  |  |  |  |  |  |
| EM79 | Positive | Type 2 diabetes | DemenTransischaemic attacks | Left hemiparesis |  |  |  |  |
| EM80 | Negative | No current active medical problem |  |  |  |  |  |  |
| EM81 | Negative | No current active medical problem |  |  |  |  |  |  |
| EM82 | Positive | No current active medical problem |  |  |  |  |  |  |
| EM83 | Negative | Depression for 40 years | Hypothyroidism | Osteoporosis |  |  |  |  |
| EM84 | Negative | Right hemiparesis (recovering) |  |  |  |  |  |  |
| EM85 | Positive | No current active medical problem |  |  |  |  |  |  |
| EM86 | Positive | Low Hb (query cause) |  |  |  |  |  |  |
| EM87 | Positive | Type 2 diabetes - requires insulin | Ischaemic heart disease and stenting | Diabetic peripheral neuropathy | Lumbar disc surgery | Gastritis and upper GI bleed | Mild cognitive impairment | Anxiety adjustment reaction |
| EM_ABX_1 | Negative | No active medical problems at time of visit |  |  |  |  |  |  |
| EM_ABX_2 | Negative | No current active problems |  |  |  |  |  |  |
| EM_ABX_3 | Negative | Left hip intertrochanteric fracture |  |  |  |  |  |  |
| EM_ABX_4 | Negative | Addison’s disease for 30 years | Pernicious anaemia for 25 years | Underactive thyroid for 25 years | Arthritis in both knees | Osteoporosis | Raynard’s disease |  |
| EM_ABX_5 | Positive | 'dizzy episodes' |  |  |  |  |  |  |
| EM_ABX_7 | Positive | transient global amnesia (2 episodes in past year) |  |  |  |  |  |  |
| EM_ABX_8 | Negative | Pain in lower back | Pain in left leg |  |  |  |  |  |
| EM_ABX_9 | Negative | No current active medical problem |  |  |  |  |  |  |
| EM_ABX_10 | Negative | Asthma for 5 years with associated bronchitis | Pernicious anaemia | Hiatus hernia with heartburn and discomfort |  |  |  |  |
| EM_ABX_11 | Negative | Gum and sinus infection |  |  |  |  |  |  |
| EM_ABX_12 | Negative | No current active problem |  |  |  |  |  |  |
| EM_ABX_13 | Negative | No current active problem |  |  |  |  |  |  |
| EM_ABX_14 | Negative | Chest infection |  |  |  |  |  |  |
| EM_ABX_15 | Positive | Recovering from urinary tract infection |  |  |  |  |  |  |
| EM_ABX_16 | Negative | Insulin Dependent Diabetes Mellitus | Upper respiratory tract infection (URTI) | URTI's under investigation due to frequency |  |  |  |  |
| EM_ABX_17 | Negative | Chest infection | Hypertension | Unspecified Heart Problem | Constant dyspepsia due to Hiatus Hernia |  |  |  |
| EM_ABX_18 | Positive | Hypothyroidism | Depression for >20years |  |  |  |  |  |
| EM_ABX_19 | Positive | Urinary tract infection |  |  |  |  |  |  |
| EM_ABX_20 | Positive | Swollen glands on left side of neck | Hearing impaired - bilateral hearing aids |  |  |  |  |  |
