# Supplementary material for: Collateral Damage in the Human Gut Microbiome - Blastocystis Is Significantly Less Prevalent in an Antibiotic-Treated Adult Population Compared to Non-Antibiotic Treated Controls
Source: Front Cell Infect Microbiol. 2022 Feb 25;12:822475. doi: 10.3389/fcimb.2022.822475 (PMC8913940; doi:10.3389/fcimb.2022.822475)
Supplement: Supplementary file 3 [file Table_3.docx]

**Supplementary Table 3.** Overview of clinical measurements in community dwelling elderly adult groups

| **Sample Number** | **Barthel Score** | **Functional Independency Mark** | **Mini mental state exam** | **Mini Nutritional Assessment** | **Body Mass Index** | **Calf Circumference (cm)** | **Diastolic** | **Height (cm)** | **Mid Arm Circumference (cm)** | **Pulse** | **Systolic** | **Temperature (oC)** | **Weight (kg)** |
| --- | --- | --- | --- | --- | --- | --- | --- | --- | --- | --- | --- | --- | --- |
| EM6 | 20 | 126 | 30 | 29 | 29 | 38 | 83 | 153 | 32 | 56 | 160 | 34.7 | 68 |
| EM10 | 20 | 126 | 30 | 27 | 24 | 32 | 69 | 174.5 | 26 | 76 | 129 | 35.6 | 72 |
| EM11 | 20 | 126 | 28 | 26 | 42.5 | 46 | 79 | 151 | 40 | 64 | 165 | 36.5 | 97 |
| EM12 | 20 | 126 | 30 | 29 | 23 | 32 | 82 | 163 | 27 | 72 | 127 | 36.2 | 62 |
| EM14 | 20 | 126 | 30 | 26 | 27.8 | 39 | 63 | 182 | 29 | 57 | 125 | 36.2 | 92 |
| EM15 | 20 | 126 | 30 | 28 | 22.1 | 31 | 94 | 174 | 28 | 63 | 158 | 35.9 | 67 |
| EM16 | 20 | 125 | 29 | 24 | 23.2 | 31 | 90 | 175 | 26 | 72 | 165 | 36 | 71 |
| EM17 | 19 | 124 | 30 | 27 | 30.4 | 34 | 76 | 155 | 32 | 69 | 135 | 36.7 | 73.1 |
| EM19 | 20 | 125 | 30 | 28.5 | 25.2 | 37 | 78 | 163 | 28 | 78 | 127 | 36.6 | 67 |
| EM22 | 20 | 126 | 30 | 26 | 25 | 31.5 | 77 | 153 | 25 | 62 | 150 | 35.9 | 58 |
| EM25 | 20 | 125 | 30 | 28 | 31.2 | 39 | 76 | 156 | 30 | 65 | 136 | 35 | 76 |
| EM26 | 20 | 125 | 29 | 26 | 26.7 | 35 | 73 | 173 | 27 | 68 | 124 | 36.3 | 80 |
| EM27 | 20 | 125 | 30 | 28.5 | 25.2 | 39 | 81 | 170 | 29 | 78 | 131 | 35.8 | 72.7 |
| EM28 | 20 | 126 | 30 | 28 | 25 | 36 | 92 | 170 | 26 | 72 | 176 | 35.5 | 72 |
| EM29 | 20 | 125 | 28 | 27 | 30.6 | 40 | 96 | 178 | 30 | 82 | 190 | 36.3 | 97 |
| EM30 | 20 | 126 | 30 | 27 | 25.7 | 34 | 77 | 159 | 28 | 76 | 152 | 35.8 | 65 |
| EM32 | 20 | 126 | 29 | 25 | 31.6 | 36 | 95 | 152 | 29 | 84 | 125 | 37 | 73 |
| EM33 | 20 | 126 | 28 | 28 | 24.9 | 36 | 90 | 164 | 26 | 66 | 160 | 35.5 | 67 |
| EM34 | 20 | 125 | 29 | 23.5 | 29 | 35.5 | 72 | 176 | 28 | 57 | 136 | 36.3 | 90 |
| EM35 | 20 | 126 | 30 | 28 | 27 | 35 | 67 | 168 | 29 | 63 | 126 | 35 | 76.1 |
| EM37 | 20 | 126 | 30 | 28.5 | 22.9 | 36 | 72 | 166 | 27 | 64 | 124 | 36 | 63 |
| EM38 | 20 | 126 | 29 | 27 | 31.2 | 36 | 73 | 164 | 31 | 78 | 148 | 36.7 | 84 |
| EM39 | 20 | 125 | 27 | 28 | 35 | 48 | 83 | 175 | 35 | 77 | 149 | 35.6 | 107.6 |
| EM40 | 20 | 125 | 30 | 28 | 28.3 | 36 | 75 | 165 | 28 | 79 | 164 | 35 | 77 |
| EM41 | 20 | 125 | 30 | 28 | 27.4 | 40 | 61 | 172 | 30 | 50 | 131 | 36 | 81 |
| EM42 | 20 | 126 | 29 | 27.5 | 28 | 37 | 72 | 164 | 25 | 62 | 144 | 35.7 | 75 |
| EM43 | 20 | 126 | 29 | 27.5 | 30 | 38 | 92 | 155 | 26 | 68 | 157 | 35.8 | 72 |
| EM44 | 17 | 121 | 27 | 26 | 29.4 | 39 | 70 | 170 | 30 | 72 | 129 | 36.2 | 85 |
| EM45 | 20 | 126 | 30 | 26.5 | 30 | 42 | 90 | 180 | 31 | 74 | 145 | 36.1 | 105 |
| EM46 | 17 | 115 | 25 | 26.5 | 24 | 33 | 61 | 173 | 26 | 74 | 159 | 36.2 | 72 |
| EM47 | 19 | 125 | 29 | 29 | 26.9 | 35.5 | 74 | 164 | 29 | 66 | 124 | 36.6 | 72.4 |
| EM48 | 16 | 109 | 27 | 26.5 | 28.7 | 38 | 60 | 175 | 28 | 89 | 106 | 36.1 | 88 |
| EM49 | 18 | 125 | 29 | 28 | 27 | 34 | 64 | 167 | 28 | 82 | 140 | 36.2 | 75 |
| EM50 | 20 | 126 | 27 | 28 | 24.6 | 35 | 72 | 177 | 29 | 72 | 168 | 36.1 | 77 |
| EM51 | 20 | 126 | 29 | 28 | 21.6 | 35 | 74 | 161 | 28 | 66 | 132 | 37 | 55.8 |
| EM52 | 20 | 125 | 30 | 26 | 21.5 | 35 | 72 | 160 | 23 | 87 | 110 | 35.8 | 55 |
| EM53 | 20 | 126 | 30 | 26.5 | 27.5 | 36 | 73 | 160 | 30 | 60 | 132 | 36.5 | 73 |
| EM54 | 20 | 126 | 29 | 26.5 | 31 | 40 | 80 | 170 | 30 | 64 | 147 | 36.1 | 89.6 |
| EM55 | 20 | 125 | 29 | 25 | 45 | 42 | 74 | 155 | 36 | 72 | 142 | 35.7 | 108 |
| EM56 | 20 | 125 | 27 | 28.5 | 26.3 | 35 | 98 | 156 | 29 | 88 | 164 | 36.4 | 64 |
| EM57 | 20 | 126 | 29 | 23.5 | 21 | 30 | 64 | 165 | 24 | 68 | 110 | 36.3 | 58.2 |
| EM58 | 20 | 125 | 25 | 26.5 | 30 | 40 | 77 | 160 | 32 | 78 | 129 | 36.3 | 77 |
| EM59 | 20 | 126 | 29 | 25.5 | 25.4 | 35 | 63 | 170 | 27 | 68 | 122 | 35.8 | 75 |
| EM60 | 20 | 126 | 30 | 28 | 34.1 | 46 | 76 | 186 | 34 | 72 | 129 | 35.9 | 118 |
| EM61 | 20 | 126 | 28 | 27 | 28.3 | 34 | 102 | 150 | 28 | 72 | 164 | 37 | 68 |
| EM62 | 20 | 125 | 22 | 27 | 24.2 | 34 | 68 | 164 | 24 | 62 | 160 | 36.4 | 65 |
| EM63 | 20 | 126 | 27 | 28 | 20.8 | 34 | 82 | 155 | 24 | 81 | 123 | 35.7 | 50 |
| EM64 | 20 | 126 | 29 | 25.5 | 22 | 34 | 80 | 164 | 25 | 72 | 161 | 36.3 | 59.4 |
| EM65 | 20 | 126 | 27 | 26.5 | 21.3 | 34 | 76 | 168 | 24 | 65 | 165 | 35.6 | 60 |
| EM66 | 20 | 126 | 29 | 23 | 19.5 | 31 | 76 | 160 | 26 | 68 | 142 | 35.5 | 50.4 |
| EM67 | 20 | 125 | 21 | 28 | 27.1 | 37 | 67 | 164 | 28 | 107 | 139 | 35.8 | 73 |
| EM68 | 20 | 125 | 29 | 27.5 | 25.7 | 34 | 86 | 165 | 28 | 86 | 137 | 36.1 | 70 |
| EM69 | 20 | 125 | 25 | 24 | 27 | 32 | 71 | 163 | 29 | 68 | 135 | 35.6 | 71.6 |
| EM70 | 20 | 124 | 27 | 27 | 27.8 | 33 | 72 | 157 | 31 | 72 | 176 | 35.5 | 68.5 |
| EM71 | 20 | 125 | 29 | 26.5 | 23.6 | 34 | 62 | 158 | 23 | 71 | 167 | 35.9 | 59 |
| EM72 | 20 | 124 | 29 | 28 | 29.4 | 36 | 96 | 165 | 29 | 78 | 162 | 36.6 | 80 |
| EM73 | 20 | 126 | 29 | 25 | 27.2 | 37 | 90 | 185 | 29 | 71 | 162 | 35.9 | 93 |
| EM74 | 20 | 126 | 29 | 27.5 | 31.7 | 42 | 88 | 173 | 31 | 74 | 166 | 36.2 | 95 |
| EM75 | 20 | 126 | 28 | 25 | 24 | 36 | 65 | 167 | 26 | 62 | 143 | 35.8 | 66 |
| EM76 | 20 | 126 | 28 | 27.5 | 31.3 | 40 | 60 | 175 | 30 | 68 | 143 | 36.2 | 96 |
| EM77 | 20 | 126 | 27 | 26 | 24 | 34 | 80 | 154 | 25 | 79 | 125 | 36.1 | 57 |
| EM78 | 20 | 125 | 28 | 26 | 24 | 35 | 74 | 175 | 25 | 65 | 142 | 36 | 74 |
| EM79 | 20 | 126 | 30 | 27.5 | 23 | 32 | 82 | 157 | 24 | 72 | 126 | 36 | 57 |
| EM80 | 20 | 126 | 26 | 24.5 | 41 | 50 | 89 | 165 | 39 | 85 | 153 | 36.6 | 111 |
| EM81 | 20 | 126 | 29 | 27.5 | 25 | 39 | 68 | 163 | 30 | 83 | 131 | 36.1 | 67 |
| EM82 | 20 | 126 | 28 | 28 | 47.7 | 45 | 78 | 158 | 31 | 60 | 125 | 36 | 119 |
| EM83 | 20 | 124 | 28 | 27.5 | 27 | 34 | 77 | 156 | 28 | 88 | 127 | 35.9 | 65 |
| EM84 | 20 | 124 | 26 | 26 | 32 | 36 | 87 | 153 | 29 | 75 | 165 | 36.2 | 75 |
| EM85 | 20 | 126 | 30 | 26.5 | 24 | 36 | 67 | 160 | 26 | 72 | 125 | 35.9 | 61 |
| EM86 | 20 | 125 | 30 | 26.5 | 20.3 | 32 | 62 | 157 | 21 | 72 | 155 | 35.6 | 50 |
| EM87 | 20 | 125 | 27 | 22.5 | 30.5 | 35 | 68 | 181 | 29 | 68 | 149 | 36.5 | 100 |
| EM88 | 20 | 126 | 29 | 29 | 24.7 | 34 | 80 | 156 | 26 | 72 | 151 | 36 | 60 |
| EM_ABX_5 | 20 | 125 | 28 | 26 | 25.5 | 31 | 77 | 150 | 26 | 78 | 138 | 36.1 | 59 |
| EM_ABX_7 | 20 | 125 | 28 | 24.5 | 25 | 38 | 73 | 180 | 27 | 85 | 120 | 36.3 | 82 |
| EM_ABX_8 | 20 | 125 | 27 | 24.5 | 30.6 | 32 | 94 | 148 | 30 | 92 | 181 | 35.8 | 67 |
| EM_ABX_9 | 20 | 126 | 28 | 27.5 | 22.8 | 33 | 77 | 151 | 23 | 72 | 130 | 35.7 | 52 |
| EM_ABX_10 | 20 | 126 | 29 | 27.5 | 30.5 | 32 | 98 | 146 | 32 | 72 | 153 | 36.3 | 65 |
| EM_ABX_11 | 20 | 126 | 29 | 26.5 | 21.3 | 33 | 78 | 172 | 25 | 93 | 120 | 36.5 | 63 |
| EM_ABX_12 | 20 | 124 | 29 | 26.5 | 26.5 | 34 | 67 | 159 | 28 | 91 | 123 | 36.6 | 67 |
| EM_ABX_13 | 20 | 126 | 29 | 28 | 28.5 | 42 | 91 | 183 | 35 | 70 | 149 | 35.6 | 95.5 |
| EM_ABX_14 | 20 | 126 | 29 | 25.5 | 40.9 | 41 | 88 | 158 | 39 | 84 | 139 | 36 | 102 |
| EM_ABX_15 | 20 | 125 | 27 | 24.5 | 30.7 | 38 | 81 | 152 | 28 | 71 | 141 | 35.4 | 71 |
| EM_ABX_16 | 20 | 120 | 13 | 29 | 29.4 | 38 | 86 | 165 | 33 | 88 | 152 | 36.5 | 80 |
| EM_ABX_17 | 20 | 126 | 28 | 28 | 29.8 | 34 | 83 | 168 | 31 | 85 | 149 | 36.4 | 84 |
| EM_ABX_18 | 14 | 100 | 28 | 22.5 | 26.6 | 33 | 86 | 161 | 30.5 | 76 | 171 | 36.3 | 69 |
| EM_ABX_19 | 20 | 126 | 30 | 28 | 28 | 38.5 | 71 | 160 | 31 | 77 | 123 | 36 | 72 |
| EM_ABX_20 | 20 | 124 | 25 | 26.5 | 21.2 | 30.5 | 73 | 155 | 22.5 | 78 | 130 | 36.8 | 51 |
| EM_ABX_21 | 20 | 126 | 29 | 28 | 32.3 | 39 | 74 | 165 | 32 | 69 | 153 | 36.7 | 88 |
| EM_ABX_22 | 20 | 126 | 26 | 28 | 25.3 | 33 | 73 | 165 | 32 | 82 | 152 | 36.6 | 69 |
| EM_ABX_23 | 16 | 96 | 19 | 18 | 20.1 | 28.5 | 96 | 161 | 24 | 98 | 145 | 36.9 | 52 |
| EM_ABX_24 | 20 | 126 | 26 | 26 | 28.7 | 35.5 | 72 | 155 | 31 | 67 | 167 | 36.6 | 69 |
| EM_ABX_25 | 18 | 115 | 23 | 28 | 30.3 | 34 | 88 | 153 | 32 | 86 | 166 | 36.8 | 71 |
| EM_ABX_26 | 20 | 126 | 26 | 23 | 16 | 32 | 78 | 158 | 20 | NA | 160 | 36.6 | 40 |
| EM_ABX_27 | 18 | 105 | 28 | 24.5 | 31.6 | 38 | 84 | 165 | 30 | 69 | 124 | 36.6 | 86 |
| EM_ABX_28 | 20 | 126 | 27 | 27 | 28.3 | 38.5 | 97 | 168 | 32 | 68 | 166 | 36.6 | 80 |
| EM_ABX_29 | 20 | 126 | 29 | 27 | 28 | 36 | 76 | 158 | 28 | 60 | 140 | 36.8 | 70 |
| EM_ABX_30 | 20 | 126 | 30 | 27.5 | 30 | 39 | 76 | 175 | 35.5 | 60 | 152 | 35.2 | 92 |
| EM_ABX_31 | 20 | 126 | 29 | 27.5 | 29.5 | 35.5 | 85 | 153 | 31 | 48 | 135 | 36.6 | 69 |
| EM_ABX_33 | 20 | 126 | 25 | 28 | 25.8 | 31.5 | 73 | 155 | 29 | 73 | 160 | 35.8 | 62 |
| EM_ABX_34 | 20 | 126 | 30 | 26 | 20.3 | 31 | 69 | 157 | 25.5 | 64 | 152 | 35 | 50 |
| EM_ABX_35 | 19 | 119 | 27 | 26.5 | 23.5 | 36 | 76 | 165 | 26 | 63 | 105 | 36.1 | 64 |
| EM_ABX_36 | 18 | 121 | 27 | 22 | 32.5 | 40 | 86 | 171 | 42 | Not done | 148 | 35.9 | 95 |
| EM_ABX_37 | 18 | 117 | 26 | 27.5 | 27.4 | 41 | 71 | 175 | 30 | 54 | 153 | 35 | 84 |
| EM_ABX_38 | 20 | 126 | 27 | 28 | 29 | 41.5 | NA | 163 | 37 | 67 | NA | 36.7 | 77 |
| EM_ABX_39 | 20 | 126 | 26 | 21 | 29.6 | 34 | 80 | 158 | 30 | 72 | 146 | 36.2 | 74 |
| EM_ABX_40 | 20 | 126 | 26 | 24 | 30.8 | 36.5 | 75 | 158 | 34 | 72 | 154 | 36.5 | 77 |
| EM_ABX_41 | 18 | 121 | 29 | 23 | 30.4 | 36.5 | 65 | 155 | 30 | 61 | 146 | 36.6 | 73 |
| EM_ABX_42 | 20 | 126 | 22 | 25.5 | 30.6 | 37 | NA | 179 | 33 | 70 | NA | 36.8 | 98 |
| EM_ABX_43 | 20 | 126 | 28 | 27 | 36.9 | 48.5 | 82 | 158 | 35.5 | 57 | 166 | 36.7 | 92 |
| EM_ABX_44 | 20 | 126 | 29 | 22 | 30.5 | 35.5 | 70 | 165 | 28.5 | 64 | 132 | 36.7 | 83 |
| EM_ABX_45 | 20 | 126 | 24 | 27.5 | 32.6 | 35 | 75 | 168 | 32.5 | 68 | 137 | 36.8 | 92 |
| EM_ABX_46 | 20 | 126 | 30 | 27 | 28.3 | 39 | 86 | 168 | 32 | 66 | 144 | 36.6 | 80 |
| EM_ABX_47 | 20 | 126 | 26 | 22 | 16 | 28 | 96 | 158 | 22 | 85 | 168 | 35.4 | 40 |
| EM_ABX_48 | 20 | 126 | 28 | 23 | 33.1 | 47 | 79 | 165 | 34 | 51 | 143 | 35.6 | 90 |
| EM_ABX_49 | 20 | 126 | 28 | 29 | 25.6 | 30 | 88 | 171 | 30 | 82 | 131 | 36.5 | 75 |
| EM_ABX_50 | 20 | 126 | 25 | 24.5 | 40.3 | 41 | 80 | 163 | 46 | 79 | 140 | 35.3 | 107 |
| EM_ABX_51 | 20 | 126 | 30 | 28 | 33.4 | 40 | 80 | 165 | 33.5 | 78 | 127 | 36.9 | 91 |
| EM_ABX_52 | 20 | 126 | 30 | 25.5 | 21.3 | 33.5 | 82 | 160 | 23.5 | 68 | 138 | 36.8 | 54.5 |
| EM_ABX_53 | 20 | 126 | 17 | 22 | 33.3 | 40.5 | 78 | 168 | 33.5 | 80 | 167 | 36.8 | 94 |
| EM_ABX_54 | 9 | 70 | 25 | 20.5 | 26.7 | 38 | 73 | 171 | 29 | 51 | 158 | 36.4 | 78 |
| EM_ABX_55 | 20 | 126 | 29 | 26.5 | 32.5 | 39 | 94 | 171 | 34 | 71 | 188 | 36.8 | 95 |
| EM_ABX_56 | 17 | 86 | 27 | 25 | 28.3 | 36 | 95 | 168 | 30 | 62 | 193 | 36.4 | 80 |
| EM_ABX_57 | 20 | 126 | 28 | 27 | 32 | 39 | 79 | 165 | 33 | 52 | 161 | 36.9 | 87 |
| EM_ABX_58 | 16 | 108 | 27 | 27.5 | 29.7 | 34 | 71 | 162 | 34 | 79 | 144 | 35.5 | 78 |
| EM_ABX_59 | 20 | 126 | 26 | 25 | 25.4 | 33 | 73 | 160 | 26.5 | 69 | 162 | 36.1 | 65 |
| EM_ABX_60 | 20 | 126 | 25 | 28.5 | 30.1 | 41 | 87 | 159 | 31 | 75 | 145 | 36.5 | 76 |
| EM_ABX_61 | 20 | 125 | 30 | 26.5 | 32.3 | 40.5 | 92 | 165 | 35 | 62 | 157 | 35.8 | 88 |
| EM_ABX_62 | 20 | 126 | 25 | 26.5 | 26.9 | 40 | 92 | 168 | 31 | 69 | 159 | 35 | 76 |
| EM_ABX_63 | 20 | 126 | 24 | 25 | 27.5 | 36 | 82 | 155 | 28 | 80 | 127 | 36.2 | 66 |
| EM_ABX_64 | 20 | 126 | 29 | 23 | 21.6 | 34.5 | 78 | 155 | 26 | 63 | 132 | 36.4 | 52 |
| EM_ABX_65 | 20 | 126 | 30 | 27.5 | 28.7 | 36.5 | 82 | 167 | 28 | 57 | 138 | 36.6 | 80 |
| EM_ABX_66 | 20 | 126 | 22 | 25.5 | 26.7 | 35 | 90 | 150 | 28 | 78 | 168 | 36.6 | 60 |
| EM_ABX_67 | 20 | 126 | 28 | 19 | 19.9 | 29.5 | 82 | 160 | 23 | 88 | 172 | 36.4 | 51 |
| EM_ABX_68 | 20 | 126 | 23 | 23 | 32.5 | 38.5 | 82 | 153 | 31 | 68 | 135 | 36.5 | 76 |
| EM_ABX_69 | 20 | 126 | 23 | 25 | 18.4 | 31 | 71 | 168 | 25 | 56 | 166 | 36 | 52 |
| EM_ABX_70 | 20 | 126 | NA | 21.5 | 37.7 | 38 | 73 | 152 | 32 | 79 | 120 | 36.9 | 87 |
| EM_ABX_71 | 20 | 126 | 27 | 26 | 32.5 | 40 | 95 | 171 | 36 | 76 | 165 | 35.7 | 95 |
| EM_ABX_72 | 20 | 126 | 29 | 27.5 | 25.7 | 37 | 90 | 165 | 32 | 64 | 144 | 36 | 70 |
| EM_ABX_73 | 20 | 126 | 25 | 25.5 | 27.4 | 34 | 75 | 175 | 28 | 80 | 145 | 36.6 | 84 |
| EM_ABX_74 | 20 | 126 | 28 | 28 | 27.2 | 37 | 86 | 165 | 32 | 74 | 137 | 36.8 | 74 |
| EM_ABX_75 | 20 | 126 | 28 | 24.5 | 28.1 | 36 | 72 | 175 | 31.5 | 51 | 144 | 36.9 | 86 |
| EM_ABX_76 | 20 | 126 | 26 | 24.5 | 25.8 | NA | 67 | 175 | 29 | 59 | 120 | 36.6 | 79 |
| EM_ABX_77 | 20 | 126 | 24 | 24 | 21.5 | 32.5 | 92 | 160 | 26 | 72 | 182 | 36.2 | 55 |
| EM_ABX_78 | 20 | 126 | 25 | 26 | 26 | 32 | 76 | 1.58 | 27 | 80 | 178 | 36.8 | 65 |
| EM_ABX_79 | 20 | 126 | 26 | 18 | 24.1 | 33 | 96 | 155 | 24 | 115 | 140 | 36.8 | 58 |
| EM_ABX_80 | 20 | 126 | 29 | 24 | 23.2 | 34 | 75 | 162 | 29 | 75 | 140 | 36.7 | 67 |
| EM_ABX_81 | 20 | 126 | 28 | 23.5 | 28.7 | 34.5 | 77 | 155 | 29.5 | 83 | 145 | 36.8 | 69 |
| EM_ABX_82 | 20 | 126 | 24 | 26 | 26.8 | 41 | 89 | 178 | 27.5 | 58 | 176 | 36.7 | 85 |
| EM_ABX_83 | 20 | 126 | 29 | 21.5 | 31.9 | NA | 59 | 167 | 30.5 | 59 | 121 | 36.2 | 89 |
| EM_ABX_84 | 20 | 126 | 27 | 25 | 24.5 | 33 | 71 | 175 | 29 | 76 | 142 | 36.2 | 75 |
| EM_ABX_85 | 16 | 111 | 28 | 16 | 23 | 33 | 77 | 1.71 | 24 | 88 | 146 | 36.4 | 67 |
| EM_ABX_86 | 20 | 126 | 28 | 23 | 26.7 | 34.5 | 64 | 1.71 | 27 | 54 | 136 | 36.2 | 78 |
| EM_ABX_87 | 20 | 126 | 29 | 26.5 | 25.5 | 34 | 88 | 1.68 | 27 | 101 | 160 | 36.7 | 72 |

NA: Not available
