# Supplementary material for: Collateral Damage in the Human Gut Microbiome - Blastocystis Is Significantly Less Prevalent in an Antibiotic-Treated Adult Population Compared to Non-Antibiotic Treated Controls
Source: Front Cell Infect Microbiol. 2022 Feb 25;12:822475. doi: 10.3389/fcimb.2022.822475 (PMC8913940; doi:10.3389/fcimb.2022.822475)
Supplement: Supplementary file 4 [file Table_4.docx]

**Supplementary Table 4.** Overview of antibiotic classes and treatments in community dwelling antibiotic-treated elderly adult group

| **Sample Code** | **Date of visit** | **On ABX within 1 month of visit** |  | **Current and previous antibiotic use** | | | **Date of Stool sample** | **Number of days since last ABX treatment** | **Comment** | ***Blastocystis* Result** | **ABX grouping** | **No. ABXs taken in recent history** |  |
| --- | --- | --- | --- | --- | --- | --- | --- | --- | --- | --- | --- | --- | --- |
|  |  |  | **ABX Class** | **ABX name (most recent first)** | **ABX dose** | **ABX**  **- dates** |  |  |  |  |  |  |  |
| EM_ABX_1 | 22/01/2009 | Yes | Penicillin | Augmentin duo | 400mgs BD (IV) | 1/1/2009 - 7/1/2009 | 22/01/2009 | 15 days |  | Negative | CWSI | 1 |  |
| EM_ABX_2 | 03/03/2009 | Yes | Penicillin | Clonamox | 250 Mgs TID | 16/02/2009 - 23/02/2009 | 03/03/2009 | 8 days |  | Negative | CWSI | 1 |  |
| EM_ABX_3 | 03/03/2009 | Yes | Azole antifungal | Fluconazole | 50mgs | 01/02/2009 - 14/02/2009 | 03/03/2009 | 17 days | recurrent chest infections & pneumonia | Negative | CWSI, NAI and PSI | 5 |  |
|  |  |  | Penicillin | Tazocin | 4.5g TDS (IV) | 22/10/2008 - 29/10/2008 |  |  |  |  |  |  |  |
|  |  |  | Macrolide | Clarithromycin | 500mgs BD | 22/10/2008 - 27/10/2008 |  |  |  |  |  |  |  |
|  |  |  | Semi-synthetic glycopeptide | Teicoplanin | 400mgs BD (IV) | 22/10/2008 - 24/10/2008 |  |  |  |  |  |  |  |
|  |  |  | Nitroimidazole | Metronidazole | 500mgs TDS | 22/10/2008 - 24/10/2008 |  |  |  |  |  |  |  |
| EM_ABX_4 | 10/03/2009 | Yes | Fluoroquinolone | Ciprofloxacin | 250mgs x 2 daily | 03/03/2009 - 07/03/2009 | 10/03/2009 | 2 days |  | Negative | NAI | 1 |  |
| EM_ABX_5 | 09/06/2009 | Yes | Penicillin | Co-amoxicillin | 625mgs | 06/05/2009-15/05/2009 | 09/06/2009 | 25 days |  | Positive | CWSI | 1 |  |
| EM_ABX_7 | 31/08/2009 | Yes | Macrolide | Klacid | 250mgs x 2 daily | 27/07/2009 - 02/08/2009 | 31/08/2009 | 29 days |  | Positive | PSI | 1 |  |
| EM_ABX_8 | 07/10/2009 | Yes | Cephalosporin | Distaclor LA | 375mgs BD | 11/09/2009 | 07/10/2009 | 26 days |  | Negative | CWSI | 1 |  |
| EM_ABX_9 | 21/10/2009 | Yes | Tetracycline | Tetracycline | 300mgs x 4 daily | 28/09/2009-04/10/2009 | 21/10/2009 | 17 days |  | Negative | PSI | 1 |  |
| EM_ABX_10 | 27/04/2010 | Yes | Penicillin | Clavamel forte | 500mgs bd | 06/04/2010-11/04/2010 | 27/04/2010 | 16 days |  | Negative | CWSI | 1 |  |
| EM_ABX_11 | 17/05/2010 | Yes | Macrolide | Klacid LA | 500mgs od | 28/04/2010-04/05/2010 | 17/05/2010 | 13 days |  | Negative | PSI | 3 |  |
|  |  |  | Macrolide | Clarithromycin | 500mgs bd | 16/12/2009-22/12/2009 |  |  |  |  |  |  |  |
|  |  |  | Macrolide | Clarithromycin | 500mgs bd | 02/10/2009-08/10/2009 |  |  |  |  |  |  |  |
| EM_ABX_12 | 06/07/2010 | Yes | Macrolide | Klacid LA | 500mgs od | 27/05/2010 - 07/06/2010 | 07/07/2010 | 29 days |  | Negative | PSI and CWSI | 2 |  |
|  |  |  | Penicillin | Augmentin Duo | 625mgs bd | 25/05/2010 - 31/05/2010 |  |  |  |  |  |  |  |
| EM_ABX_13 | 04/08/2010 | Yes | Macrolide | Klacid | 250mgs bd | 12/07/2010-18/07/2010 | 04/08/2010 | 17 days |  | Negative | PSI and CWSI | 2 |  |
|  |  |  | Penicillin | Amoxicillin | 250mgs tds | 23/06/2010-29/06/2010 |  |  |  |  |  |  |  |
| EM_ABX_14 | 23/08/2010 | Yes | Penicillin | Flucloxacillin | 500mgs tds | 07/08/2010-due to finish 20/8/2010 | 18/08/2010 | ongoing | Chest infection | Negative | CWSI | 5 |  |
|  |  |  | Penicillin | Flucloxacillin | 500mgs qds | Flucloxacillin 31/07/2010-06/08/2010 |  |  |  |  |  |  |  |
|  |  |  | Cephalosporin | Zinnat | 500mgs bd | 31/03/2010-06/04/2010 |  |  |  |  |  |  |  |
|  |  |  | Penicillin | Flucloxacillin | 500mgs qds | 22/02/2010-28/02/2010 |  |  |  |  |  |  |  |
|  |  |  | Penicillin | Calvapen | 666mgs qds | 22/02/2010-28/02/2010 |  |  |  |  |  |  |  |
| EM_ABX_15 | 08/09/2010 | Yes | Trimethoprim | Trimethoprim | 200mgs bd | 27/08/2010-31/08/2010 | 08/09/2010 | 8 days | Recovering from urinary tract infection | Positive | CWSI and NAI | 3 |  |
|  |  |  | Penicillin | Amoxycillin | 500mgs tds | 06/08/2010-12/08/2010 |  |  |  |  |  |  |  |
|  |  |  | Penicillin | Amoxycillin | 500mgs tds | 11/04/2010-17/04/2010 |  |  |  |  |  |  |  |
| EM_ABX_16 | 23/09/2010 | Yes | Penicillin | Clavamel | 625mgs BD | 13/09/10-19/09/10 | 23/09/2010 | 4 days | Upper respiratory tract infection (URTI) | Negative | CWSI and NAI | 8 |  |
|  |  |  | Penicillin | Clavamel | 375mgs TDS | 19/08/10-28/08/10 |  |  |  |  |  |  |  |
|  |  |  | Cephalosporin | Velosef | 500mgs BD | 10/06/10-14/06/10 |  |  |  |  |  |  |  |
|  |  |  | Cephalosporin | Distaclor LA | 375mgs BD | 11/05/10-15/05/10 |  |  |  |  |  |  |  |
|  |  |  | Fluoroquinolone | Taravid | one tab BD | 31/03/10-06/04/10 |  |  |  |  |  |  |  |
|  |  |  | Cephalosporin | Cifox | 250mgs BD | 15/02/10-19/02/10 |  |  |  |  |  |  |  |
|  |  |  | Trimethoprim – cell wall inhibition | Trimethoprim | 100mgs BD | 31/12/09-04/01/10 |  |  |  |  |  |  |  |
|  |  |  | Penicillin | Augmentin | Not Specified | 18/12/09-Not stated |  |  |  |  |  |  |  |
| EM_ABX_17 | 30/09/2010 | Yes | Penicillin | Penamox | 500mgs TDS | 20/09/10-26/09/10 | 30/09/2010 | 4 days | Chest infection | Negative | CWSI | 1 |  |
| EM_ABX_18 | 20/10/2010 | Yes | Penicillin | Augmentin Duo | 625mgs BD PO | 04/10/10 - 11/10/10 | 20/10/2010 | 9 days |  | Positive | CWSI | 1 |  |
| EM_ABX_19 | 29/11/2011 | Yes | Penicillin | Augmentin duo | 625mgs bd | 16/11/2010-22/11/2010 | 28/11/2010 | 6 days | Urinary tract infection | Positive | CWSI and NAI | 7 |  |
|  |  |  | Cephalosporin | Ceflex | 250mgs qds | 08/11/2010-12/11/2010 |  |  |  |  |  |  |  |
|  |  |  | Cephalosporin | Distaclor LA | 375mgs bd | 31/08/2010-04/09/2010 |  |  |  |  |  |  |  |
|  |  |  | Nitrofurantoin | Macrodantin | 400mgs daily | 04/03/2010-08/03/2010 |  |  |  |  |  |  |  |
|  |  |  | Cephalosporin | Cefaclor | 250mgs tds | 21/12/2009-25/12/2009 |  |  |  |  |  |  |  |
|  |  |  | Fluoroquinolone | Ciprofloxacin | 500mgs bd | 02/12/2009-06/12/2009 |  |  |  |  |  |  |  |
|  |  |  | Azole antifungal | Difflucan | 150mgs (single dose) | 09/01/2009 |  |  |  |  |  |  |  |
| EM_ABX_20 | 30/11/2010 | Yes | Penicillin | Augmentin Duo | 625mgs TDS | 22/11/2010 - 24/11/2010 | 03/12/2010 | 9 days | Swollen glands, shingles (3 weeks ago) | Positive | CWSI | 4 |  |
|  |  |  | Famvir (antiviral) | Famvir (antiviral) | 750mgs OD | 17/11/2010 - 24/11/2010 |  |  |  |  |  |  |  |
|  |  |  | Penicillin | Augmentin Duo | 625mgs BD | 17/11/2010 - 22/11/2010 |  |  |  |  |  |  |  |
|  |  |  | Penicillin | Flucloxacillin | 250mgs QDS | 02/11/2010 - 08/11/2010 |  |  |  |  |  |  |  |
| EM_ABX_21 | 08/02/2011 | Yes | Penicillin | Augmentin Duo | 625mgs TDS | 10/02/2011 - due to finish 16/02/2011 | 11/02/2011 | ongoing | Chest infection | Negative | CWSI | 2 |  |
|  |  |  | Penicillin | Augmentin Duo | 625mgs TDS | 30/01/2011 - 05/02/2011 |  |  |  |  |  |  |  |
| EM_ABX_22 | 09/02/2011 | Yes | Trimethoprim – cell wall inhibition | Trimethoprim | 100mgs OD | 10/06/2010 - ongoing prophylactic antibiotic | 09/02/2011 | ongoing | Recurrent UTIs | Negative | CWSI and NAI | 12 |  |
|  |  |  | Cephalosporin | Distaclor LA | 375mgs BD | 17/01/2011 - 21/01/2011 |  |  |  |  |  |  |  |
|  |  |  | Penicillin | Augmentin Duo | 625mgs BD | 16/12/2010 - 20/12/2010 |  |  |  |  |  |  |  |
|  |  |  | Penicillin | Augmentin Duo | 625mgs BD | 02/07/2010 - 08/07/2010 |  |  |  |  |  |  |  |
|  |  |  | Nitrofurantoin | Macrodantin | 50mgs QDS | 27/05/2010 - 02/06/2010 |  |  |  |  |  |  |  |
|  |  |  | Penicillin | Augmentin Duo | 625mgs BD | 17/05/2010 - 21/05/2010 |  |  |  |  |  |  |  |
|  |  |  | Cephalosporin | Velosef | 250mgs TDS | 30/04/2010 - 09/05/2010 |  |  |  |  |  |  |  |
|  |  |  | Penicillin | Augmentin Duo | 625mgs BD | 26/04/2010 - 05/05/2010 |  |  |  |  |  |  |  |
|  |  |  | Penicillin | Augmentin Duo | 625mgs BD | 09/04/2010 - 13/04/2010 |  |  |  |  |  |  |  |
|  |  |  | Penicillin | Pinaclav | 375mgs TDS | 18/03/2010 - 22/03/2010 |  |  |  |  |  |  |  |
|  |  |  | Cephalosporin | Distaclor LA | 375mgs BD | 15/02/2010 - 19/02/2010 |  |  |  |  |  |  |  |
|  |  |  | Penicillin | Augmentin Duo | 625mgs BD | 08/02/2010 - 14/02/2010 |  |  |  |  |  |  |  |
| EM_ABX_23 | 14/02/2011 | Yes | Macrolide | Klarithromycin | 500mgs BD | 26/01/2011 - 01/02/2011 | 14/02/2011 | 13 days | Bronchitis, chest infection | Negative | CWSI,PSI and NAI | 4 |  |
|  |  |  | Cephalosporin | Distaclor LA | 375mgs BD | 08/12/2010 - 14/12/2010 |  |  |  |  |  |  |  |
|  |  |  | Penicillin | Clonamox | 250mgs TDS | 22/11/2010 - 28/11/2010 |  |  |  |  |  |  |  |
|  |  |  | Nitrofurantoin | Macrodantin | 100mgs OD | 01/03/2010 - 31/03/2010 |  |  |  |  |  |  |  |
| EM_ABX_24 | 22/02/2011 | Yes | Penicillin | Geriflox | 500mgs TDS | 14/02/2011 - 20/02/2011 | 22/02/2011 | 2 days |  | Negative | CWSI | 1 |  |
| **EM_ABX_25** | 23/02/2011 | Yes | Penicillin | Augmentin | 375mgs TDS | 31/01/2011- 06/02/2011 | 23/02/2011 | 17 days |  | Negative | CWSI | 6 |  |
|  |  |  | Penicillin | Augmentin | 375mgs TDS | 05/01/2011 - 11/01/2011 |  |  |  |  |  |  |  |
|  |  |  | Cephalosporin | Distaclor LA | 375mgs BD | 20/10/2010 - 24/10/2010 |  |  |  |  |  |  |  |
|  |  |  | Penicillin | Geriflox | 250mgs TDS | 03/09/2010 - 07/09/2010 |  |  |  |  |  |  |  |
|  |  |  | Semi-synthetic glycopeptide | Targocid | 200mgs BD | 05/04/2010 - 10/04/2010 |  |  |  |  |  |  |  |
|  |  |  | Penicillin | Augmentin Duo | 625mgs BD | 09/04/2010 - 13/04/2010 |  |  |  |  |  |  |  |
| EM_ABX_26 | 24/02/2011 | Yes | Macrolide | Klacid | 250mgs BD | 20/01/2011 - 26/01/2011 | 24/02/2011 | 29 days |  | Negative | PSI | 7 |  |
|  |  |  | Macrolide | Klacid | 500mgs OD | 07/01/2011 - 13/01/2011 |  |  |  |  |  |  |  |
|  |  |  | Macrolide | Klacid | 500mgs OD | 08/12/2010 - 14/12/2010 |  |  |  |  |  |  |  |
|  |  |  | Macrolide | Klacid | 500mgs BD | 06/10/2010 - 16/10/2010 |  |  |  |  |  |  |  |
|  |  |  | Macrolide | Klacid | 250mgs BD | 13/09/2010 - 19/09/2010 |  |  |  |  |  |  |  |
|  |  |  | Macrolide | Klacid | 250mgs BD | 08/08/2010 - 14/08/2010 |  |  |  |  |  |  |  |
|  |  |  | Macrolide | Klacid | 500mgs OD | 26/03/2010 - 01/04/2010 |  |  |  |  |  |  |  |
| EM_ABX_27 | 03/03/2011 | Yes | Penicillin | Calvepen | 333mgs QDS | 15/02/2011 - 21/02/2011 | 03/03/2011 | 10 days |  | Negative | CWSI | 16 |  |
|  |  |  | Penicillin | Geriflox | 500mgs QDS | 15/02/2011 - 21/02/2011 |  |  |  |  |  |  |  |
|  |  |  | Penicillin | Calvepen | 333mgs QDS | 29/01/2011 -04/02/2011 |  |  |  |  |  |  |  |
|  |  |  | Penicillin | Flucloxacillin | 500mgs TDS | 29/01/2011 - 04/02/2011 |  |  |  |  |  |  |  |
|  |  |  | Penicillin | Flucloxacillin | 500mgs TDS | 25/01/2011 - 29/01/2011 |  |  |  |  |  |  |  |
|  |  |  | Penicillin | Augmentin Duo | 625mgs TDS | 06/01/2011 - 11/01/2011 |  |  |  |  |  |  |  |
|  |  |  | Penicillin | Flucloxacillin | 500mgs TDS | 28/12/2010 - 03/01/2011 |  |  |  |  |  |  |  |
|  |  |  | Cephalosporin | Zinnat | 500mgs BD | 13/12/2010 - 17/12/2010 |  |  |  |  |  |  |  |
|  |  |  | Cephalosporin | Zinnat | 500mgs BD | 13/11/2010 - 19/11/2010 |  |  |  |  |  |  |  |
|  |  |  | Penicillin | Amoxil | 500mgs TDS | 01/09/2010 - 10/09/2010 |  |  |  |  |  |  |  |
|  |  |  | Cephalosporin | Zinnat | 500mgs BD | 09/08/2010 - 15/08/2010 |  |  |  |  |  |  |  |
|  |  |  | Cephalosporin | Zinnat | 500mgs BD | 28/07/2010 - 03/08/2010 |  |  |  |  |  |  |  |
|  |  |  | Penicillin | Augmentin Duo | 625mgs TDS | 15/05/2010 - 21/05/2010 |  |  |  |  |  |  |  |
|  |  |  | Cephalosporin | Zinnat | 500mgs BD | 26/04/2010 - 02/05/2010 |  |  |  |  |  |  |  |
|  |  |  | Penicillin | Geriflox | 500mgs TDS | 22/04/2010 - 26/04/2010 |  |  |  |  |  |  |  |
|  |  |  | Cephalosporin | Zinnat | 500mgs BD | 23/02/2010 - 25/02/2010 |  |  |  |  |  |  |  |
| EM_ABX_28 | 08/03/2011 | Yes | Fucithalmic eye ointment | Fucithalmic eye ointment | BD | 01/02/2011 - 05/02/2011 | 08/03/2011 | 31 days |  | Negative | CWSI | 3 |  |
|  |  |  | Penicillin | Augmentin Duo | 625mgs BD | 01/02/2011 - 05/02/2011 |  |  |  |  |  |  |  |
|  |  |  | Locabiotal nasal spray | Locabiotal nasal spray | 500mcgs 4 hourly | 19/07/2010 - 23/07/2010 |  |  |  |  |  |  |  |
| EM_ABX_29 | 10/03/2011 | Yes | Macrolide | Klacid | 500mgs OD | 11/02/2011 - 17/02/2011 | 10/03/2011 | 21 days | Chest infection | Negative | CWSI and PSI | 3 |  |
|  |  |  | Penicillin | Amoclav | 625mgs BD | 16/09/2010 - 22/09/2010 |  |  |  |  |  |  |  |
|  |  |  | Penicillin | Amoclav | 625mgs BD | 22/04/2010 - 28/04/2010 |  |  |  |  |  |  |  |
| EM_ABX_30 | 10/03/2011 | Yes | Cephalosporin | Distaclor LA | 375mgs bd | 14/02/2011-20/02/2011 | 09/03/2011 | 17 days |  | Negative | CWSI | 1 |  |
| EM_ABX_31 | 21/03/2011 | Yes | Cephalosporin | Suprax | 200mgs OD | 08/02/2011 - 17/02/2011 | 21/03/2011 | 32 days |  | Positive | CWSI | 1 |  |
|  |  |  |  |  |  |  |  |  |  |  |  |  |  |
| EM_ABX_32 | 30/03/2011 | Yes | Penicillin | Penicillin | 333mgs BD | 1994 - continuous | 30/03/2011 | ongoing |  | Negative | CWSI | 3 |  |
|  |  |  | Penicillin | Augmentin | 375mgs TDS | 11/11/2010 - 16/11/2010 |  |  |  |  |  |  |  |
|  |  |  | Penicillin | Augmentin | 375mgs TDS | 03/11/2010 - 09/11/2010 |  |  |  |  |  |  |  |
| EM_ABX_33 | 03/05/2011 | Yes | Cephalosporin | Keflex | 250mgs daily | ongoing | 03/05/2011 | ongoing |  | Negative | CWSI | 3 |  |
|  |  |  | Cephalosporin | Zinacef | 1.5g iv x 1 dose | 20/10/2010 |  |  |  |  |  |  |  |
|  |  |  | Cephalosporin | Zinacef | 750mgs iv x 2 doses | 20/10/2010-21/10/2010 |  |  |  |  |  |  |  |
| EM_ABX_34 | 05/05/2011 | Yes | Macrolides | Zithromax | 250mgs three times a week | ongoing | 05/05/2011 | ongoing |  | Negative | CWSI and NAI | 6 |  |
|  |  |  | Penicillin | Clonamox | 500mgs tds | 01/07/2010-07/07/2010 |  |  |  |  |  |  |  |
|  |  |  | Macrolide | Zithromax | Unknown dose | 29/06/2010-Unknown |  |  |  |  |  |  |  |
|  |  |  | Nitroimidazole | Flagyl | 200mgs tds | 26/06/2010-Unknown |  |  |  |  |  |  |  |
|  |  |  | Macrolide | Zithromax | Unknown dose | 26/06/2010-Unknown |  |  |  |  |  |  |  |
|  |  |  | Cephalosporin | Distaclor LA | 375mgs bd | 08/06/2010-12/06/2010 |  |  |  |  |  |  |  |
| EM_ABX_35 | 09/05/2011 | Yes | Fluoroquinolones | Ciprofloxacin | 500mgs tds | 11/04/2011-20/04/2011 | 06/05/2011 | ongoing |  | Negative | NAI and PSI | 3 |  |
|  |  |  | Macrolide | Klacid | 250mgs bd | 04/02/2011-10/02/2011 |  |  |  |  |  |  |  |
|  |  |  | Penicillin | Pinaclav | 375mgs tds | 24/01/2011-29/01/2011 |  |  |  |  |  |  |  |
| EM_ABX_38 | 24/05/2011 | Yes | Semi-synthetic penicillin | Pinamox | 500mgs TDS | 28/04/2011 - 04/05/2011 | 24/05/2011 | 16 days |  | Positive | CWSI | 2 |  |
|  |  |  | Penicillin | Augmentin Duo | 625mgs BD | ?/01/2011 - ?/01/2011 |  |  |  |  |  |  |  |
| EM_ABX_48 | 07/07/2011 | Yes | Macrolide | Klacid | 250mgs bd | 07/06/2011-14/06/2011 | 07/07/2011 | 23 days |  | Positive | CWSI and PSI | 8 |  |
|  |  |  | Penicillin | Augmentin | 625mgs bd | 04/05/2011-14/05/2011 |  |  |  |  |  |  |  |
|  |  |  | Penicillin | Augmentin | 625mgs bd | 23/02/2011-04/03/2011 |  |  |  |  |  |  |  |
|  |  |  | Penicillin | Augmentin | 625mgs bd | 15/12/2010-25/12/2010 |  |  |  |  |  |  |  |
|  |  |  | Macrolide | Klacid | 250mgs bd | 22/09/2010-29/09/2010 |  |  |  |  |  |  |  |
|  |  |  | Penicillin | Augmentin | 625mgs bd | 05/10/2010-15/10/2010 |  |  |  |  |  |  |  |
|  |  |  | Macrolide | Klacid | 250mgs bd | 22/07/2010-29/07/2010 |  |  |  |  |  |  |  |
|  |  |  | Penicillin | Augmentin | 625mgs bd | 21/06/2010-01/07/2010 |  |  |  |  |  |  |  |
| EM_ABX_56 | 13/09/2011 | Yes | Fluoroquinolone | Ciproxin | 250mgs BD | 25/08/2011 - 29/08/2011 | 13/09/2011 | 15 days | Ulcerative colitis | Positive | CWSI and NAI | 2 |  |
|  |  |  | Penicillin | Augmentin Duo | 625mgs TDS | 24/12/2010 - 25/12/2010 |  |  |  |  |  |  |  |
| EM_ABX_71 | 13/12/2011 | Yes | Cephalosporin | Zinnat | 500mgs bd | 30/11/2011-06/12/2011 | 13/12/2011 | 7 days |  | Positive | CWSI PSI and NAI | 10 |  |
|  |  |  | Macrolide | Klacid forte | 500mgs bd | 22/11/2011-29/11/2011 |  |  |  |  |  |  |  |
|  |  |  | Quinolone | Avelox | 400mgs daily | 03/11/2011-10/11/2011 |  |  |  |  |  |  |  |
|  |  |  | Penicillin | Augmentin | 625mgs tds | 29/10//2011-03/11/2011 |  |  |  |  |  |  |  |
|  |  |  | Macrolide | Klacid | 500mgs bd | 06/2011-exact dates unknown |  |  |  |  |  |  |  |
|  |  |  | Penicillin | Augmentin | 625mgs | 06/2011-exact dates unknown |  |  |  |  |  |  |  |
|  |  |  | Cephalosporin | Cefodox | 100mgs BD | 09/05/2011-14/05/2011 |  |  |  |  |  |  |  |
|  |  |  | Tetracycline | Vibramycin | 100mgs daily | 25/02/2011-04/03/2011 |  |  |  |  |  |  |  |
|  |  |  | Macrolide | Klacid | 500mgs bd | 01/2011 for 7 days |  |  |  |  |  |  |  |
|  |  |  | Penicillin | Augmentin duo | 625mgs | 01/2011 for 7 days |  |  |  |  |  |  |  |
| EM_ABX_72 | 14/12/2011 | Yes | Cephalosporin | Keflex | 500mgs tds | 17/11/2011-19/11/2011 | 14/12/2011 | 25 days |  | Positive | CWSI | 2 |  |
|  |  |  | Penicillin | Clavamel forte | 500/125mgs | 08/04/2011-12/04/2011 |  |  |  |  |  |  |  |
| EM_ABX_73 | 19/11/2011 | Yes | Quinolone | Avelox | 400mgs BD | 16/11/2011-20/11/2011 | 19/11/2011 |  | ongoing | Negative | CWSI | 4 |  |
|  |  |  | Penicillin | Augmentin | 825mgs/125mgs BD | 12/09/2011-18/09/2011 |  |  |  |  |  |  |  |
|  |  |  | Penicillin | Augmentin | 825mgs/125mgs BD | 27/06/2011-23/06/2011 |  |  |  |  |  |  |  |
|  |  |  | Penicillin | Augmentin | 825mgs/125mgs BD | 28/03/2011-03/04/2011 |  |  |  |  |  |  |  |
| EM_ABX_74 | 20/01/2012 | Yes | Penicillin | Penicillin | 500mgs TDS | 09/01/2012 - 15/01/2012 | 20/01/2012 | 5 days | UTI | Positive | CWSI and NAI | 2 |  |
|  |  |  | Fluoroquinolone | Ciprofloxacin | 500mgs BD | 05/01/2012 - 11/01/2012 |  |  |  |  |  |  |  |
| EM_ABX_75 | 24/01/2012 | Yes | Penicillin | Clavamel forte | 500/125mgs TDS | 04/01/2012 - 11/01/2012 | 24/01/2012 | 23 days | Chest infection | Negative | CWSI | 1 |  |
| EM_ABX_76 | 01/02/2012 | Yes | Macrolide | Klacid | 500mgs BD | 04/01/2012 - 10/01/2012 | 01/02/2012 | 21 days |  | Negative | PSI | 1 |  |
| EM_ABX_77 | 30/03/2012 | Yes | Cephalosporin | Suprax | 200mgs OD | 13/03/2012 - 19/03/2012 | 29/03/2012 | 10 days | Chest infection/flu like symptoms | Negative | CWSI | 5 |  |
|  |  |  | Semi-synthetic penicillin | Pinamox | 250mgs TDS | 10/02/2012 - 16/02/2012 |  |  |  |  |  |  |  |
|  |  |  | Cephalosporin | Zinnat | 250mgs TDS | 21/02/2012 - 27/02/2012 |  |  |  |  |  |  |  |
|  |  |  | Cephalosporin | Distaclor LA | 250mgs TDS | 13/01/2012 - 19/01/2012 |  |  |  |  |  |  |  |
|  |  |  | Penicillin | Fucillin | 500mgs QDS | 11/01/2012 - 17/01/2012 |  |  |  |  |  |  |  |
| EM_ABX_78 | 13/03/2012 | Yes | Trimethoprim | Trimethoprim | 500mgs BD | 27/02/2012 - 02/03/2012 | 12/03/2012 | 10 days |  | Negative | CWSI, NAI and PSI | 5 |  |
|  |  |  | Penicillin | Clavamel forte | 625mgs BD | 06/12/2011 - 12/12/2011 |  |  |  |  |  |  |  |
|  |  |  | Fluoroquinolone | Ciprofloxacin | 500mgs BD | 29/07/2011 - 04/08/2011 |  |  |  |  |  |  |  |
|  |  |  | Penicillin | Clavamel forte | 625mgs BD | 22/06/2011 - 26/06/2011 |  |  |  |  |  |  |  |
|  |  |  | Penicillin | Clavamel forte | 625mgs BD | 01/04/2011 - 10/04/2011 |  |  |  |  |  |  |  |
| EM_ABX_79 | 02/04/2012 | Yes | Macrolide | Clorom | 500mgs BD | 27/03/2012 - 05/04/2012 | 03/04/2012 | ongoing | Recurrent urinary tract infections with persistent pseudomonas / catheterised | Negative | CWSI and PSI | 2 |  |
|  |  |  | Semi-synthetic penicillin | Pinamox | 500mgs TDS | 16/03/2012 - 22/03/2012 |  |  |  |  |  |  |  |
| EM_ABX_80 | 03/04/2012 | Yes | Penicillin | Germentin | 625mgs TDS | 14/03/2012 - 18/03/2012 | 03/04/2012 | 16 days |  | Negative | CWSI and PSI | 1 |  |
| EM_ABX_81 | 05/04/2012 | Yes | Semi-synthetic penicillin | Pinamox | 500mgs TDS | 26/03/2012 - 01/04/2012 | 05/04/2012 | 4days | Chest infection |  | CWSI | 3 |  |
|  |  |  | Penicillin | Fucillin | 500mgs TDS | 21/10/2011 - 27/10/2012 |  |  |  |  |  |  |  |
|  |  |  | Penicillin | Pinaclav | 500mgs / 125mgs TDS | 02/09/2011 - 08/09/2011 |  |  |  |  |  |  |  |
| EM_ABX_82 | 23/04/2012 | Yes | Penicillin | Amoxicillin | 250mgs TDS | 20/03/2012 - 26/03/2012 | 22/04/2012 | 27 days |  | Negative | CWSI | 1 |  |
| EM_ABX_83 | 26/04/2012 | Yes | Penicillin | Amoxicillin | 500mgs QDS | 14/03/12-20/03/2012 | 26/04/2012 | 37 days |  | Negative | CWSI and NAI | 7 |  |
|  |  |  | Nitroimidazole | Flagyl | 400mgs BD | 10/02/2012-16/02/2012 |  |  |  |  |  |  |  |
|  |  |  | Penicillin | Flucillin | 500mgs BD | 10/02/2012-16/02/2012 |  |  |  |  |  |  |  |
|  |  |  | Nitroimidazole | Flagyl | 400mgs BD | 31/01/2012-05/02/2012 |  |  |  |  |  |  |  |
|  |  |  | Penicillin | Flucillin | 500mgs BD | 31/01/2012-05/02/2012 |  |  |  |  |  |  |  |
|  |  |  | Nitroimidazole | Flagyl | 400mgs BD | 05/09/2011-11/09/2011 |  |  |  |  |  |  |  |
|  |  |  | Penicillin | Flucillin | 500mgs BD | 05/09/2011-11/09/2011 |  |  |  |  |  |  |  |
| EM_ABX_84 | 04/05/2012 | Yes | Cephalosporin | Zinnat | 250mgs BD | 11/04/2012 - 17/04/2012 | 04/05/2012 | 17 days |  | Negative | CWSI | 1 |  |
| EM_ABX_85 | 09/07/2012 | Yes | Penicillin | Augmentin Duo | 500mgs TDS | 26/06/2012 - 02/07/2012 | 10/07/2012 | 8 days | Prone to chest infections | Negative | CWSI and NAI | 7 |  |
|  |  |  | Cephalosporin | Cifox | 250mgs BD | 12/04/2012 - 18/04/2012 |  |  |  |  |  |  |  |
|  |  |  | Penicillin | Augmentin Duo | 500mgs TDS | 18/01/2011 - 24/01/2011 |  |  |  |  |  |  |  |
|  |  |  | Fluoroquinolone | Ciproxin | 250mgs TDS | 19/11/2011 - 25/11/2011 |  |  |  |  |  |  |  |
|  |  |  | Penicillin | Pinaclav | 500mgs TDS | 08/11/2011 - 14/11/2011 |  |  |  |  |  |  |  |
|  |  |  | Penicillin | Augmentin Duo | 500mgs TDS | 06/09/2011 - 12/09/2011 |  |  |  |  |  |  |  |
|  |  |  | Cephalosporin | Cifox | 250mgs BD | 03/08/2011 - 06/08/2011 |  |  |  |  |  |  |  |
| EM_ABX_86 | 10/07/2012 | Yes | Penicillin | Amoxicillin | 500mgs TDS | 18/06/2012 - 24/06/2012 | 10/07/2012 | 16 days |  | Negative | CWSI | 7 |  |
|  |  |  | Macrolide | Clarithromycin | 250mgs BD | 17/04/2012 - 23/04/2012 |  |  |  |  |  |  |  |
|  |  |  | Macrolide | Clarithromycin | 250mgs BD | 19/01/2012 - 25/01/2012 |  |  |  |  |  |  |  |
|  |  |  | Penicillin | Augmentin Duo | 625mgs TDS | 10/01/2012 - 16/01/2012 |  |  |  |  |  |  |  |
|  |  |  | Penicillin | Clonamox | 500mgs TDS | 05/05/2011 - 11/05/2011 |  |  |  |  |  |  |  |
| EM_ABX_87 | 30/07/2012 | Yes | Penicillin | Augmentin Duo | 625mgs TDS | 13/07/2012 - 19/07/2012 | 30/07/2012 | 9 days |  | Negative | CWSI, NAI | 18 |  |
|  |  |  | Semi-synthetic penicillin | Pinamox | 500mgs QDS | 12/07/2012 - 21/07/2012 |  |  |  |  |  |  |  |
|  |  |  | Nitrofurantoin | Macrodantin | 50mgs QDS | 12/07/2012 - 21/07/2012 |  |  |  |  |  |  |  |
|  |  |  | Fluoroquinolones | Ciprofloxacin | 500mgs BD | 07/07/2012 - 13/07/2012 |  |  |  |  |  |  |  |
|  |  |  | Penicillin | Augmentin Duo | 625mgs TDS | 24/05/2012 - 30/05/2012 |  |  |  |  |  |  |  |
|  |  |  | Penicillin | Augmentin Duo | 625mgs TDS | 24/04/2012 - 30/04/2012 |  |  |  |  |  |  |  |
|  |  |  | Penicillin | Augmentin Duo | 625mgs TDS | 06/04/2012 - 10/04/2012 |  |  |  |  |  |  |  |
|  |  |  | Penicillin | Augmentin Duo | 625mgs TDS | 03/04/2012 - 09/04/2012 |  |  |  |  |  |  |  |
|  |  |  | Penicillin | Augmentin Duo | 625mgs TDS | 08/03/2012 - 14/03/2012 |  |  |  |  |  |  |  |
|  |  |  | Penicillin | Flucloxacillin | 500mgs TDS | 05/03/2012 - 09/03/2012 |  |  |  |  |  |  |  |
|  |  |  | Fluoroquinolone | Ciprofloxacin | 500mgs BD | 04/11/2011 - 10/11/2011 |  |  |  |  |  |  |  |
|  |  |  | Fluoroquinolone | Ciprofloxacin | 500mgs BD | 03/11/2011 - 04/11/2011 |  |  |  |  |  |  |  |
|  |  |  | Penicillin | Augmentin Duo | 625mgs TDS | 01/11/2011 - 07/11/2011 |  |  |  |  |  |  |  |
|  |  |  | Fluoroquinolone | Ciprofloxacin | 500mgs BD | 05/10/2011 - 09/10/2011 |  |  |  |  |  |  |  |
|  |  |  | Penicillin | Augmentin Duo | 625mgs TDS | 30/09/2011 - 04/10/2011 |  |  |  |  |  |  |  |
|  |  |  | Penicillin | Augmentin Duo | 625mgs TDS | 26/09/2011 - 02/10/2011 |  |  |  |  |  |  |  |
|  |  |  | Fluoroquinolone | Ciprofloxacin | 500mgs BD | 22/07/2011 - 28/07/2011 |  |  |  |  |  |  |  |
|  |  |  | Penicillin | Augmentin Duo | 625mgs TDS | 12/07/2011 - 18/07/2011 |  |  |  |  |  |  |  |

Codes: CWSI = cell wall synthesis inhibition, NAI = nucleic acid inhibition and PSI = protein synthesis inhibition
