# Supplementary material for: Collateral Damage in the Human Gut Microbiome - Blastocystis Is Significantly Less Prevalent in an Antibiotic-Treated Adult Population Compared to Non-Antibiotic Treated Controls
Source: Front Cell Infect Microbiol. 2022 Feb 25;12:822475. doi: 10.3389/fcimb.2022.822475 (PMC8913940; doi:10.3389/fcimb.2022.822475)
Supplement: Supplementary file 5 [file Table_5.docx]

**Supplementary Table 5**. Alpha diversity indices result per individual

| **Sample Number** | ***Blastocytis* PCR result** | **Chao1** | **ACE** | **Shannon** | **Simpson** |
| --- | --- | --- | --- | --- | --- |
| EM6 | Positive | 263.0526316 | 247.8987829 | 3.667873464 | 0.94789056 |
| EM10 | Positive | 363.4473684 | 369.0573484 | 4.235032742 | 0.97310568 |
| EM11 | Negative | 241.55 | 234.1688797 | 3.579026584 | 0.94332352 |
| EM12 | Positive | 276.04 | 263.3332328 | 3.741227978 | 0.91374528 |
| EM14 | Positive | 247.7307692 | 267.6884319 | 3.566615069 | 0.94571168 |
| EM15 | Negative | 154.3157895 | 163.3610912 | 3.077405988 | 0.8962504 |
| EM16 | Negative | 220.3448276 | 227.9267449 | 2.83176864 | 0.80267904 |
| EM17 | Positive | 368.5277778 | 367.8650097 | 4.255486645 | 0.9722092 |
| EM19 | Positive | 251.5555556 | 260.8136012 | 3.615228226 | 0.94643272 |
| EM22 | Negative | 392.6829268 | 393.6624785 | 4.322031149 | 0.96866624 |
| EM25 | Positive | 358.9574468 | 372.6371616 | 4.13051459 | 0.95935584 |
| EM26 | Negative | 372.5 | 374.5786142 | 3.963168736 | 0.95695976 |
| EM27 | Positive | 161.7142857 | 157.9369655 | 3.059230867 | 0.88458496 |
| EM28 | Negative | 195 | 195.6676705 | 3.212954726 | 0.916168 |
| EM29 | Negative | 105.25 | 112.1292924 | 2.004885911 | 0.76328416 |
| EM30 | Positive | 219.7857143 | 231.8547767 | 4.065142712 | 0.97181936 |
| EM32 | Negative | 204.3 | 204.8650932 | 3.476136168 | 0.9308928 |
| EM33 | Negative | 234.8076923 | 242.4744122 | 3.331524106 | 0.92900152 |
| EM34 | Negative | 291.0285714 | 298.6965062 | 4.001574513 | 0.96554296 |
| EM35 | Negative | 273.9375 | 258.690449 | 3.770951594 | 0.9589312 |
| EM37 | Positive | 371.6666667 | 359.054527 | 4.34985715 | 0.97029576 |
| EM38 | Negative | 412.1627907 | 419.8499561 | 4.103228887 | 0.95921936 |
| EM39 | Positive | 296.3030303 | 300.8985534 | 3.690939096 | 0.93983696 |
| EM40 | Positive | 276.7567568 | 290.384783 | 3.483638337 | 0.9027976 |
| EM41 | Positive | 208.0526316 | 205.5655787 | 3.829532702 | 0.95305624 |
| EM42 | Positive | 337 | 299.5943995 | 3.606754783 | 0.90110376 |
| EM43 | Negative | 260.3448276 | 264.3404894 | 3.480638143 | 0.93850768 |
| EM44 | Negative | 307.3571429 | 294.5045148 | 3.878106487 | 0.934212 |
| EM45 | Positive | 372.5 | 342.7068388 | 3.935817293 | 0.93709384 |
| EM46 | Negative | 95.15789474 | 105.0354945 | 2.711338055 | 0.84086928 |
| EM47 | Positive | 319.25 | 344.9735781 | 3.918108841 | 0.96294584 |
| EM48 | Negative | 290.9166667 | 277.931834 | 3.662035448 | 0.95040184 |
| EM49 | Negative | 343.15 | 355.3345804 | 4.128222487 | 0.96850976 |
| EM50 | Positive | 245.7142857 | 241.2507814 | 3.815168548 | 0.94955624 |
| EM51 | Positive | 357.8709677 | 344.2932941 | 4.060425505 | 0.94170352 |
| EM52 | Negative | 253.2 | 271.3694553 | 3.627309547 | 0.95064176 |
| EM53 | Positive | 419 | 399.7482884 | 4.19089013 | 0.96977552 |
| EM54 | Positive | 338.5416667 | 307.9656939 | 4.048636451 | 0.96950408 |
| EM55 | Negative | 163.05 | 167.9941506 | 3.09815815 | 0.88847368 |
| EM56 | Positive | 332 | 338.3656319 | 3.853234497 | 0.9449312 |
| EM57 | Positive | 245.0416667 | 241.3543808 | 3.960679011 | 0.96654256 |
| EM58 | Positive | 339.5416667 | 305.0367831 | 3.795871862 | 0.9505148 |
| EM59 | Positive | 291.0909091 | 292.0144916 | 3.952498618 | 0.96458536 |
| EM60 | Negative | 299.8333333 | 295.9790863 | 3.829154696 | 0.95421544 |
| EM61 | Negative | 258.0909091 | 270.2334289 | 3.211937023 | 0.86690872 |
| EM62 | Negative | 274.4347826 | 262.9112843 | 3.680791307 | 0.95846464 |
| EM63 | Positive | 279.5555556 | 283.8802725 | 4.023079638 | 0.95655872 |
| EM64 | Negative | 402.375 | 378.8134701 | 4.336838811 | 0.97250552 |
| EM65 | Positive | 379.25 | 359.2636922 | 3.923683585 | 0.9171788 |
| EM66 | Negative | 193.0769231 | 176.8291127 | 2.929586129 | 0.87273256 |
| EM67 | Negative | 355.3333333 | 363.5481088 | 3.923764393 | 0.95977752 |
| EM68 | Positive | 382.5882353 | 377.979078 | 4.060494375 | 0.95580848 |
| EM69 | Negative | 302.7894737 | 262.5430971 | 3.752288433 | 0.95260608 |
| EM70 | Positive | 306.5 | 315.7292028 | 4.225044697 | 0.97571824 |
| EM71 | Negative | 294 | 303.0732699 | 4.027967552 | 0.96357648 |
| EM72 | Positive | 283.0384615 | 272.1409194 | 3.966713949 | 0.95708152 |
| EM73 | Positive | 342.9655172 | 346.2624211 | 4.155397052 | 0.97043 |
| EM74 | Negative | 198.2 | 194.0716705 | 3.438073852 | 0.93779696 |
| EM75 | Positive | 275.1153846 | 275.2835469 | 3.481154146 | 0.8965268 |
| EM76 | Positive | 265 | 243.8466622 | 3.403336332 | 0.90522256 |
| EM77 | Positive | 344 | 331.0277798 | 3.84196638 | 0.95631624 |
| EM78 | Positive | 234.5769231 | 250.1778382 | 3.368435894 | 0.91825936 |
| EM79 | Positive | 225.1351351 | 246.3359183 | 3.328970949 | 0.91019976 |
| EM80 | Negative | 172.3333333 | 163.3723537 | 3.227066657 | 0.90901776 |
| EM81 | Negative | 327.5 | 310.0999105 | 3.774381501 | 0.93275752 |
| EM82 | Positive | 347.7142857 | 307.1945271 | 4.079178178 | 0.96908608 |
| EM83 | Negative | 233.3333333 | 221.2620684 | 3.425712528 | 0.908384 |
| EM84 | Negative | 208 | 205.9330836 | 3.49776123 | 0.91792568 |
| EM85 | Positive | 455.0555556 | 359.6894897 | 4.52229583 | 0.98192584 |
| EM86 | Positive | 298.9032258 | 296.00873 | 3.9025868 | 0.96040368 |
| EM87 | Positive | 289.8846154 | 299.3390777 | 3.087450226 | 0.89461248 |
| EM88 | Positive | 328.7575758 | 333.6353383 | 3.883381016 | 0.95695984 |
| EM_ABX_5 | Positive | 326.7727273 | 295.2060758 | 3.711646239 | 0.937102 |
| EM_ABX_7 | Positive | 377.8846154 | 349.4433474 | 4.528211715 | 0.98183224 |
| EM_ABX_8 | Negative | 125.4 | 146.0043235 | 2.332174905 | 0.82418328 |
| EM_ABX_9 | Negative | 227.8 | 219.8581036 | 3.66880828 | 0.95473672 |
| EM_ABX_10 | Negative | 236.6875 | 218.4986988 | 3.3944334 | 0.93253424 |
| EM_ABX_11 | Negative | 287.6896552 | 286.9887978 | 3.368258447 | 0.9237144 |
| EM_ABX_12 | Negative | 357.2380952 | 379.497477 | 4.084032436 | 0.96681848 |
| EM_ABX_13 | Negative | 246.5652174 | 272.8863786 | 3.412612065 | 0.92000824 |
| EM_ABX_14 | Negative | 161.3333333 | 163.9566246 | 2.24296516 | 0.82495432 |
| EM_ABX_15 | Positive | 215.0416667 | 220.3810836 | 3.09952431 | 0.8965856 |
| EM_ABX_16 | Negative | 264.4285714 | 296.1594597 | 2.480643945 | 0.79020528 |
| EM_ABX_17 | Negative | 287.2413793 | 286.0779213 | 3.528536554 | 0.92106504 |
| EM_ABX_18 | Positive | 319.516129 | 334.746362 | 3.521896014 | 0.91772112 |
| EM_ABX_19 | Positive | 199.7142857 | 202.7603382 | 3.139131387 | 0.91195872 |
| EM_ABX_20 | Positive | 262.8 | 263.8391255 | 3.077660768 | 0.89126584 |
| EM_ABX_21 | Negative | 265.125 | 260.7572235 | 3.280262591 | 0.92219912 |
| EM_ABX_22 | Negative | 262.75 | 279.6558319 | 3.2254627 | 0.92511512 |
| EM_ABX_23 | Negative | 243.037037 | 262.3046626 | 2.969749299 | 0.89617072 |
| EM_ABX_24 | Negative | 279.625 | 270.3243891 | 3.211119851 | 0.90664888 |
| EM_ABX_25 | Negative | 233.2068966 | 247.4306756 | 3.619629347 | 0.94203968 |
| EM_ABX_26 | Negative | 301.5416667 | 294.0830026 | 3.451464467 | 0.93133248 |
| EM_ABX_27 | Negative | 233.03125 | 264.9099381 | 3.424099109 | 0.94253288 |
| EM_ABX_28 | Negative | 194.6818182 | 207.6039618 | 3.080683014 | 0.90486048 |
| EM_ABX_29 | Negative | 99.86666667 | 112.4409433 | 2.109449017 | 0.75462808 |
| EM_ABX_30 | Negative | 262.1764706 | 235.0776224 | 3.726445844 | 0.95324352 |
| EM_ABX_31 | Positive | 216.6470588 | 203.6465625 | 3.365630153 | 0.92826072 |
| EM_ABX_33 | Negative | 378.6071429 | 346.8950961 | 4.209280271 | 0.97316312 |
| EM_ABX_34 | Negative | 231.2727273 | 196.6560086 | 3.12294722 | 0.90993128 |
| EM_ABX_35 | Negative | 196.6818182 | 210.2574291 | 3.05305182 | 0.91086248 |
| EM_ABX_36 | Negative | 120.2 | 98.23258631 | 1.987542673 | 0.76233768 |
| EM_ABX_37 | Negative | 240.8666667 | 223.7776636 | 3.40479437 | 0.9302176 |
| EM_ABX_38 | Positive | 374.7837838 | 376.2906258 | 4.293068565 | 0.97036728 |
| EM_ABX_39 | Negative | 138.0769231 | 136.1902778 | 2.951166806 | 0.89334872 |
| EM_ABX_40 | Negative | 296.1052632 | 274.1623761 | 3.696450599 | 0.94224152 |
| EM_ABX_41 | Negative | 138.1538462 | 154.0414633 | 2.918851926 | 0.9153868 |
| EM_ABX_42 | Negative | 290.1714286 | 302.5339578 | 3.512457076 | 0.9393656 |
| EM_ABX_43 | Negative | 204.4736842 | 205.0469939 | 3.359242954 | 0.92652304 |
| EM_ABX_44 | Negative | 210.7894737 | 206.8210361 | 3.670536049 | 0.9567884 |
| EM_ABX_45 | Negative | 223.2173913 | 230.4292939 | 3.419006396 | 0.92914416 |
| EM_ABX_46 | Negative | 138.6470588 | 137.0717462 | 2.749158131 | 0.83869632 |
| EM_ABX_47 | Negative | 243.7692308 | 205.4125224 | 3.212024365 | 0.90229944 |
| EM_ABX_48 | Positive | 269.8076923 | 269.280691 | 3.853249381 | 0.95667952 |
| EM_ABX_49 | Negative | 264.9 | 247.7844184 | 3.865252208 | 0.95890008 |
| EM_ABX_50 | Negative | 215.04 | 224.168239 | 3.300098232 | 0.89359944 |
| EM_ABX_51 | Negative | 216.047619 | 212.7310746 | 3.583433948 | 0.9469572 |
| EM_ABX_52 | Negative | 229.0434783 | 226.4923108 | 3.762237078 | 0.95198176 |
| EM_ABX_53 | Negative | 245.8 | 257.4522635 | 3.385734155 | 0.91796544 |
| EM_ABX_54 | Negative | 362.1 | 267.7672044 | 3.355593042 | 0.89879376 |
| EM_ABX_55 | Negative | 199 | 205.1632384 | 3.574841607 | 0.94787968 |
| EM_ABX_56 | Positive | 299.5 | 323.4101379 | 3.774574279 | 0.95477888 |
| EM_ABX_57 | Negative | 161.0769231 | 163.9773718 | 2.182749578 | 0.76035832 |
| EM_ABX_58 | Negative | 214.0555556 | 218.0823848 | 3.264987004 | 0.92003088 |
| EM_ABX_59 | Negative | 191.2307692 | 175.8708801 | 3.459121511 | 0.94205248 |
| EM_ABX_60 | Negative | 196.1428571 | 199.6185186 | 3.426197931 | 0.94220872 |
| EM_ABX_61 | Negative | 397.2 | 360.8721372 | 4.18238972 | 0.96880088 |
| EM_ABX_62 | Negative | 239.0857143 | 274.7873851 | 3.323003839 | 0.92435576 |
| EM_ABX_63 | Negative | 143 | 142.4250186 | 2.442769412 | 0.7861608 |
| EM_ABX_64 | Negative | 234.64 | 255.6974896 | 2.966719736 | 0.87944304 |
| EM_ABX_65 | Negative | 332.2162162 | 345.0260135 | 3.184571668 | 0.82623496 |
| EM_ABX_66 | Negative | 299.5384615 | 303.1616633 | 3.189441806 | 0.91590696 |
| EM_ABX_67 | Negative | 375.7777778 | 375.2660318 | 4.0917318 | 0.96778496 |
| EM_ABX_68 | Negative | 149.6666667 | 121.0426041 | 2.700892949 | 0.8769824 |
| EM_ABX_69 | Negative | 327.5 | 309.6224669 | 3.579141769 | 0.94061488 |
| EM_ABX_70 | Negative | 80.23529412 | 83.28879921 | 2.071446342 | 0.72409064 |
| EM_ABX_71 | Positive | 259.7727273 | 245.2103469 | 3.49464253 | 0.93974272 |
| EM_ABX_72 | Positive | 227.55 | 224.115353 | 3.162420012 | 0.90051392 |
| EM_ABX_73 | Negative | 219.1071429 | 235.8495763 | 3.183202572 | 0.8985876 |
| EM_ABX_74 | Positive | 197.5555556 | 206.008957 | 3.330078845 | 0.9366728 |
| EM_ABX_75 | Negative | 295.5769231 | 298.9577882 | 3.723884785 | 0.95497128 |
| EM_ABX_76 | Negative | 197.84 | 204.7898515 | 3.349933462 | 0.91566368 |
| EM_ABX_77 | Negative | 302.2413793 | 285.9805872 | 3.667579266 | 0.93884768 |
| EM_ABX_78 | Negative | 175.7307692 | 198.3911499 | 2.864256978 | 0.84310464 |
| EM_ABX_79 | Negative | 162.1764706 | 160.8813424 | 2.098725936 | 0.68769112 |
| EM_ABX_80 | Negative | 254.2142857 | 254.3335102 | 3.152497506 | 0.88831312 |
| EM_ABX_81 | Negative | 135.0625 | 159.2778584 | 2.681029685 | 0.86978248 |
| EM_ABX_82 | Negative | 329.0909091 | 330.2051739 | 4.095140915 | 0.96139224 |
| EM_ABX_83 | Negative | 253 | 240.5364892 | 3.585889946 | 0.946054 |
| EM_ABX_84 | Negative | 171.7142857 | 164.7278397 | 2.957098985 | 0.91217208 |
| EM_ABX_85 | Negative | 182 | 201.5052641 | 2.875802939 | 0.8541396 |
| EM_ABX_86 | Positive | 292.8947368 | 279.7700234 | 3.211108474 | 0.89720248 |
| EM_ABX_87 | Negative | 214.2 | 201.067711 | 3.47181863 | 0.92762096 |
