# Supplementary material for: Collateral Damage in the Human Gut Microbiome - Blastocystis Is Significantly Less Prevalent in an Antibiotic-Treated Adult Population Compared to Non-Antibiotic Treated Controls
Source: Front Cell Infect Microbiol. 2022 Feb 25;12:822475. doi: 10.3389/fcimb.2022.822475 (PMC8913940; doi:10.3389/fcimb.2022.822475)
Supplement: Supplementary file 6 [file Table_6.docx]

**Supplementary Table 6.** Significant taxonomic associations between specific bacteria genera and *Blastocystis*

| **Genus** | **Base Mean** | **log2 Fold-Change** | **log2 FC Standard Error** | **Statistic** | **P-value** | **BH Adjusted P-Value** | **Blastocystis Positive median.** | **Blastocystis Positive inter-quartile range** | **Blastocystis Negative Median.** | **Blastocystis Negative Inter-quartile range** |
| --- | --- | --- | --- | --- | --- | --- | --- | --- | --- | --- |
| **Bacteroides↓** | 7050.146781 | -0.98 | 0.185 | -5.307 | 0.000000112 | 0.0000127 | 4134.82 | [2183.98-6045.28] | 6934.51 | [4191.04-11079.78] |
| **Bilophila ↓** | 13.227 | -1.78 | 0.38 | -4.709 | 0.00000249 | 0.000105 | 3.37 | [0.89-7.67] | 8.31 | [1.07-21.39] |
| **Escherichia/Shigella ↓** | 19.89 | -2.25 | 0.48 | -4.688 | 0.00000275 | 0.000105 | 0.93 | [0-3.9] | 6.66 | [0.82-37] |
| **Clostridium_XlVb ↓** | 41.5008 | -1.38 | 0.3 | -4.598 | 0.0000043 | 0.000121 | 14.92 | [6.35-28.85] | 21.77 | [8.58-57.19] |
| **Flavonifractor ↓** | 49.3232 | -1.22 | 0.298 | -4.114 | 0.000039 | 0.00089 | 10.35 | [6.6-22.88] | 21.13 | [8.68-68.22] |
| **Subdoligranulum ↓** | 23.301 | -1.23 | 0.32 | -3.847 | 0.00012 | 0.00227 | 6.29 | [3.25-10.16] | 9.03 | [4.31-24.9] |
| **Parasutterella ↓** | 132.2167 | -1.51 | 0.46 | -3.305 | 0.00095 | 0.0155 | 23.4 | [4.52-60.32] | 34.19 | [3.92-238.6] |
| **Streptococcus ↓** | 37.4434 | -1.09 | 0.36 | -3.04 | 0.0024 | 0.0274 | 10.54 | [4.76-21.49] | 13.29 | [3.43-55.55] |
| **Anaerosporobacter ↑** | 5.614 | 1.57 | 0.48 | 3.264 | 0.0011 | 0.0156 | 1.94 | [0-19.7] | 0.54 | [0-3.36] |
| **Robinsoniella ↑** | 20.958 | 1.63 | 0.51 | 3.202 | 0.00136 | 0.0173 | 14.11 | [2.41-59.06] | 0.93 | [0-12.84] |
